# Quantification and structure–function analysis of calpain-1 and calpain-2 protease subunit interactions

**DOI:** 10.1016/j.jbc.2025.110243

**Published:** 2025-05-16

**Authors:** Ivan Shapovalov, Prawin Rimal, Pitambar Poudel, Victoria Lewtas, Mathias Bell, Shailesh Kumar Panday, Brian J. Laight, Danielle Harper, Stacy Grieve, George S. Baillie, Kazem Nouri, Peter L. Davies, Emil Alexov, Peter A. Greer

**Affiliations:** 1 Department Pathology and Molecular Medicine, School of Medicine, Queen's University, Kingston, Ontario, Canada; 2 Division of Cancer Biology and Genetics, Sinclair Cancer Research Institute, Queen's University, Kingston, Ontario, Canada; 3 Department of Physics, College of Science, Clemson University, Clemson, South Carolina, USA; 4 Department of Biomedical and Molecular Sciences, Queen's University, Kingston, Ontario, Canada; 5 School of Cardiovascular and Metabolic Health, University of Glasgow, Glasgow, Scotland, UK; 6 Department of Pathology and Laboratory Medicine, University of British Columbia, Vancouver, British Columbia, Canada

**Keywords:** calpain, PEF domain, protein–protein interaction, dissociation constant, binding free energy, calcium, dimerization, allosteric regulation, biosensor

## Abstract

Calpain-1 and calpain-2 are heterodimeric proteases consisting of a common small regulatory subunit CAPNS1 and a large catalytic subunit, CAPN1 or CAPN2, respectively. These calpains have emerged as potential therapeutic targets in cancer and other diseases through their roles in cell signaling pathways affecting sensitivity to chemotherapeutic and targeted drugs and in promoting metastasis. While inhibition of calpains has the potential to provide therapeutic benefit to cancer patients, there are currently no clinically approved active site–directed drugs that specifically and effectively inhibit them. However, the structures of calpain-1 and calpain-2 make them susceptible to allosteric inhibition aimed at interfering with heterodimerization of the catalytic and regulatory subunits, which is necessary for stability and proteolytic activity. Split-Nanoluciferase biosensors were generated to quantify the protein–protein interactions between the calcium-binding penta-EF-hand domains of CAPN1 or CAPN2 and CAPNS1. These biosensors were used to quantify the heterodimer dissociation constants (*K*_*D*_) of calpain-1 and calpain-2, estimated at 185 nM and 509 nM, respectively, in the presence of 5 mM Ca^2+^; and 362 nM and 1651 nM, respectively, in the presence of Mg^2+^. The half-maximal Ca^2+^ concentrations supporting these protein–protein interactions for calpain-1 and calpain-2 were 59.9 μM and 940.8 μM, respectively. Molecular modeling, based on the crystal structure of calpain-2, was used to predict 20 residues of the penta-EF-hand domains that contribute to heterodimerization. Individual point mutation of CAPNS1 at Q263 reduced the catalytic activity of calpain-2 to 51.0 ± 6.4% in live cells.

Calpain-1 and calpain-2 are the prototypic members of the calpain family of calcium (Ca^2+^)-regulated papain-like thiol proteases (1). These ubiquitously expressed classical members of the calpain family are heterodimers consisting of two subunits: the isoform-specific CAPN1 or CAPN2 catalytic subunit, respectively; and the CAPNS1 common regulatory subunit (Fig. 1*A*) (2, 3). They have been implicated in multiple forms of cancer where high levels of expression in tumors have been correlated with poor clinical outcomes (4, 5); and evidence from preclinical mouse models has shown that genetic disruption of these two calpains in cancer cells can suppress their tumorigenic and metastatic behaviors (5, 6, 7). In addition to roles in cancer, dysregulated calpain activity has been implicated in neurodegenerative and cardiovascular diseases, muscular dystrophy, diabetes, and fibrosis; thus stimulating interest in developing clinically effective inhibitors for use in multiple indications (8, 9). While many calpain inhibitors have been described with reported activity in *in vitro* or *in vivo* models, there are currently no calpain inhibitors approved for clinical application. Available calpain inhibitors lack high degrees of specificity—in part, because most efforts have been directed at the active site, which shares extensive structural homology with many of the other ∼600 proteases encoded by the human genome (10). This prompts exploration of other modes of calpain-specific inhibition.

**Figure 1:**
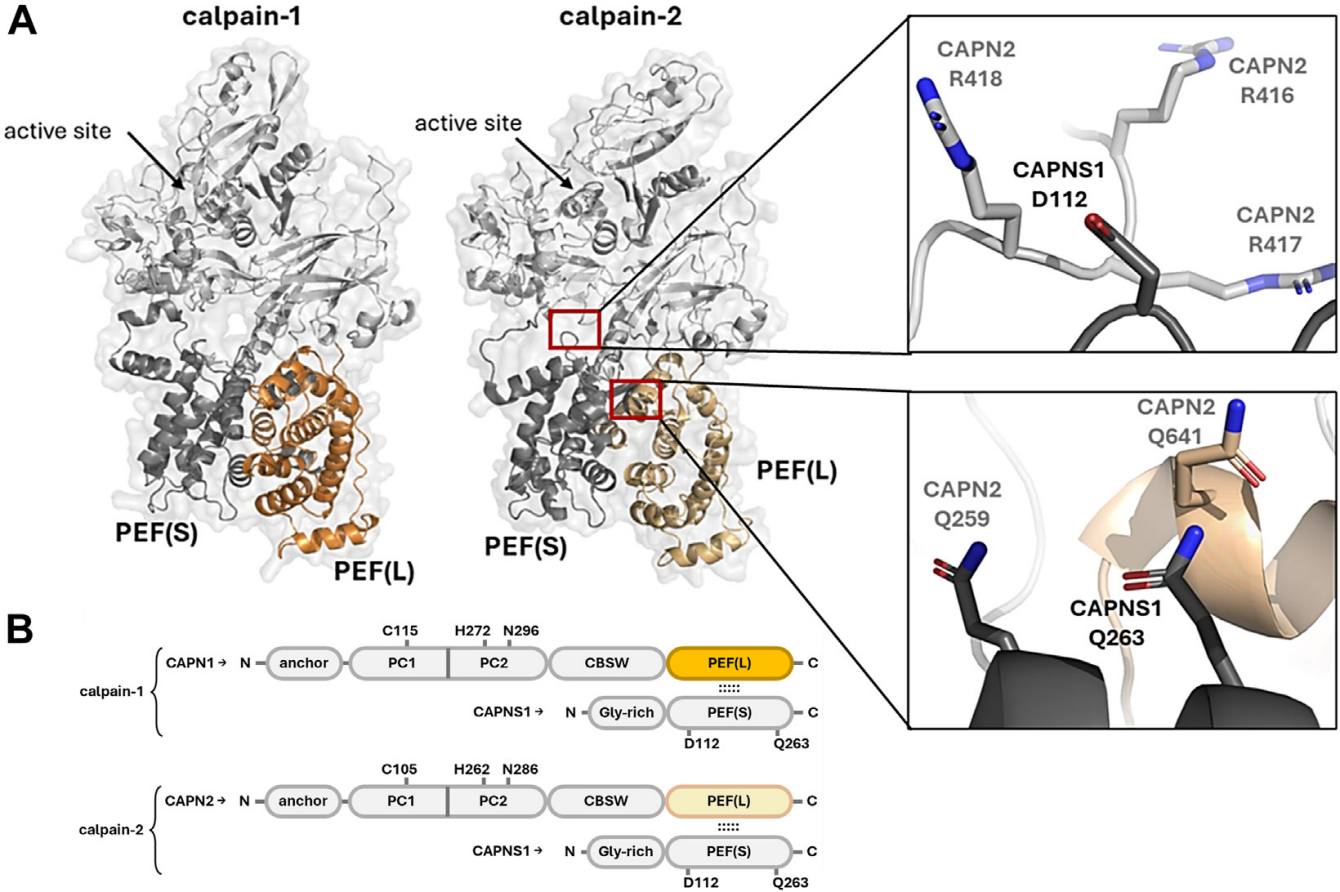
**Structures of calpain-1 and calpain-2.***A,* 3D models of calpain-1 and -2 with the active site, PEF domains, and positions of CAPNS1 D112 and Q263 highlighted. The model of human calpain-1 is based on an AlphaFold3 prediction (with the glycine-rich domain of CAPNS1 omitted) (64). The model of human calpain-2 is based on a crystal structure (Protein Data Bank ID: 1KFU) (21). *B,* domain illustrations of calpain-1 and -2 show the N-terminal anchor helix, PC1 and PC2 protease core subdomains, CBSW calpain-type beta-sandwich domain, PEF domains in the CAPN1/2 catalytic large subunits (PEF[L]) and the CAPNS1 regulatory small subunit (PEF[S]), and the N-terminal glycine-rich domain (Gly-rich) of CAPNS1. Amino acids of the calpain-1 and -2 catalytic triads (C105/118, H262/272, and N286/296, respectively) and putative PEF–PEF PPI mediating residues D112 and Q263 in CAPNS1 are indicated. PEF, penta-EF-hand domain; PPI, protein–protein interaction.

Homologous penta-EF-hand (PEF) domains mediate heterodimerization of the regulatory CAPNS1 (small subunit—S) with either catalytic CAPN1 or CAPN2 (large subunit—L) to form the functional calpain-1 or calpain-2 enzymes, respectively (illustrated in Fig. 1*B*). The obligate nature of this PEF(S)–PEF(L) interaction for calpain-1 and calpain-2 proteases was demonstrated by genetic disruption of the *CAPNS1* gene, which abolished both isoforms; by not only preventing formation of the active heterodimeric proteases, but also by destabilizing the CAPN1 and CAPN2 catalytic subunits, leading to their degradation (11, 12). This has inspired efforts aimed at allosterically inhibiting calpain-1 and calpain-2 with small molecules capable of interfering with this PEF(S)–PEF(L) interaction (9, 13, 14, 15, 16). PEF domains are rare in the human proteome, with the EFh_PEF superfamily consisting of only five proteins other than the classical calpains (3, 17). It follows that PEF(S)–PEF(L) protein–protein interaction (PPI) inhibitors may therefore be less prone to off-target effects than active site–directed inhibitors.

The importance of Ca^2+^ to calpain activity has been well explored (1); and structural studies of calpain-1 and calpain-2 have determined how Ca^2+^ binding at two sites in their catalytic domains is associated with conformational changes resulting in alignment of the catalytic triad residues (18, 19). In addition to those two Ca^2+^-binding sites in the catalytic core, classical calpains-1 and -2 also have five Ca^2+^ binding EF hand helix–loop–helix motifs in each of the PEF domains of the CAPN1, CAPN2, and CAPNS1 subunits; and four of these five EF hands in each subunit bind Ca^2+^ (2, 20). It has been suggested that Ca^2+^ binding to the PEF domain of CAPNS1 may promote proteolytic activation *via* displacing the N-terminal anchor helix of the catalytic subunit and allowing it to be cleaved (2, 21, 22, 23); but in general, the significance of the eight EF hand Ca^2+^-binding sites for calpain dimerization and activity is not well characterized.

To measure the PEF–PEF PPIs, we have used the split-nanoluciferase NanoBiT biosensor system (24). In this system, two halves of the deep-sea shrimp luciferase, LgBiT and SmBiT, are expressed in fusion with the proteins of interest, and the PPI of those proteins is detected through the bioluminescence activity of the consequently reconstituted NanoBiT. The benefits of the split-luciferase NanoBiT system over other nonluciferase reporters include the low inherent affinity of LgBiT to SmBiT (*K*_*D*_ = 190 μM), which minimizes false-positive PPIs; and increased brightness and stability of the bioluminescent signal relative to that of luciferases from other species, which provides a more robust readout in biochemical assays (24).

In this study, we expressed SmBiT as fusion proteins with the PEF domain of either CAPN1, CAPN2, or CAPNS1, and LgBiT as a fusion protein with the PEF(S) domain of CAPNS1. After determining optimal configurations and interaction conditions, we used this system to measure heterodimerization between CAPNS1 and either CAPN1 or CAPN2 or homodimerization of CAPNS1; and performed structure–function studies exploring residues or motifs of the PEF domains, which contribute significantly to the PEF(S)–PEF(L) PPI.

To inform biochemical studies, we employed *in silico* approaches to make predictions about the importance of specific residues or surfaces in the PEF domains for calpain-2 heterodimerization. This was made possible by the availability of high-resolution crystal structures for human calpain-2 (21). This involved a multifaceted computational mutational analysis (based on machine learning methods shown to be the most accurate) (25) of the CAPN2 PEF(L) and CAPNS1 PEF(S) domains based on a calpain-2 crystal structure (Protein Data Bank [PDB] ID: 1KFU) (21); in which we calculated the effects of individual amino acid substitutions on the free energy change (ΔΔG) of the heterodimer. These *in silico* predictions were then validated by testing high-ranking mutations in the NanoBiT PEF(L)–PEF(S) biosensor PPI system or in *capns1* null mammalian cells transduced with CAPNS1 rescue constructs and assessing their effects on CAPN2 stabilization and proteolytic activity. This provided functional evidence for the contributions of individual amino acids to the PEF(L)–PEF(S) PPI and the identification of interfacial regions that could be targeted with small molecules to interfere with heterodimerization.

In summary, this study demonstrates a novel method to measure the PPIs between CAPNS1 and either CAPN1 or CAPN2; measures the affinity of these PEF(L)–PEF(S) associations in calpain-1 and calpain-2; and shows how perturbations of this interaction could be used to inhibit calpain activity in an allosteric manner.

## Results

### Design of split-Nanoluciferase calpain PPI biosensors

**Figure 2:**
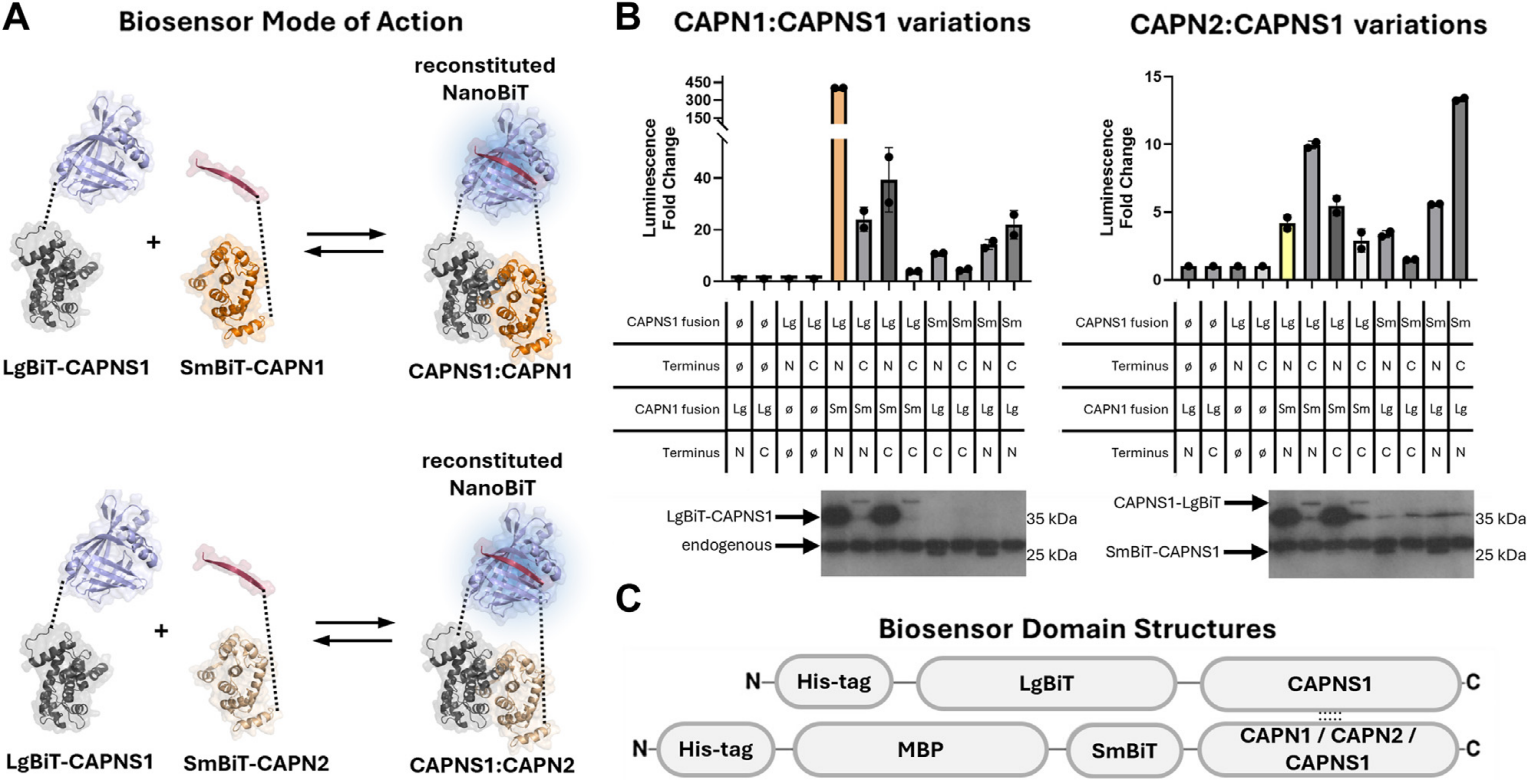
**Identification of optimal probes for detecting CAPNS1–CAPN1/2 PPIs using different orientations and combinations of PEF domains from CAPNS1 and CAPN1/2 fused with Nanoluciferase SmBiT and LgBiT fragments.** Residues 538 to 714, 524 to 700, or 60 to 268 of human CAPN1, CAPN2, or CAPNS1, respectively, were expressed as fusions with LgBiT or SmBiT pairwise in transfected HEK293T cells. The signals were defined as fold change of the luminescent output over a selected LgBiT-alone control. *A,* mechanism of the split NanoBiT PPI biosensors. *B,* luciferase signals produced with cell lysates from HEK293 cells transfected with the indicated calpain-1 (*left*) or calpain-2 (*right*) PPI biosensor orientations. Datapoints are technical replicates. Immunoblots using an antibody specific for CAPNS1 shows expression of the respective CAPNS1 fusion proteins. *C,* domain diagrams of selected optimal constructs expressed in bacteria, with oligo-histidine (His-tag) and maltose-binding protein (MBP). CAPN1/CAPN2/CAPNS1 refers only to the PEF domains of the respective proteins. HEK293T, human embryonic kidney 293T cell line; PEF, penta-EF-hand domain; PPI, protein–protein interaction.

The catalytic subunits of calpain-1 and calpain-2 heterodimerize with the common regulatory subunit through their homologous PEF domains, which consist of amino acid residues 538 to 714 in CAPN1, 524 to 700 in CAPN2, and 60 to 268 in CAPNS1. This PPI has been documented in a crystal structure of calpain-2 (21). We selected optimal split-Nanoluciferase biosensors of this PPI (Fig. 2*A*) after testing all possible orientations and combinations of fusion proteins consisting of LgBiT or SmBiT fused with each of the three PEF domains (Fig. 2*B*). Mammalian expression constructs consisting of either the LgBiT or SmBiT fused at either the N terminus or C terminus of each PEF domain (Fig. 2*B*) were coexpressed in all possible LgBiT–SmBiT PEF(S)–PEF(L) combinations in transfected human embryonic kidney 293T (HEK293T) cells. The combination of LgBiT-CAPNS1 and SmBiT-CAPN1 produced the strongest bioluminescence signal with the largest dynamic range; approximately 400-fold increase over LgBiT-CAPNS1 alone control (Fig. 2*B*, *left*). This orientation was selected for subsequent use. Although the combination of SmBiT-CAPN2 and LgBiT-CAPNS1 did not display the highest signal for the calpain-2 biosensors (Fig. 2*B*, *right*), it was selected because it was the same relative orientation as the selected calpain-1 biosensor. This allowed the use of the same LgBiT-CAPNS1 component for both biosensors, and the PEF domains were at the C terminus for all constructs. Based on this analysis, three bacterial expression constructs simulating calpain-1 and calpain-2 heterodimerization were designed to produce and purify: His_10_-LgBiT-CAPNS1, His_6_-MBP-SmBiT-CAPN1, and His_6_-MBP-SmBiT-CAPN2. In addition, the His_6_-MBP-SmBiT-CAPNS1 construct was designed, purified, and utilized to measure the calpain small subunit homodimer PPI, which may influence the stoichiometry of heterodimerization. The constructs are schematically illustrated in Figure 2*C*. The His-tag enabled Ni^2+^ chelate affinity purification, and maltose-binding protein (MBP) enabled optimal soluble expression in bacteria.

### Production and assay optimization of purified calpain PPI biosensor components

**Figure 3:**
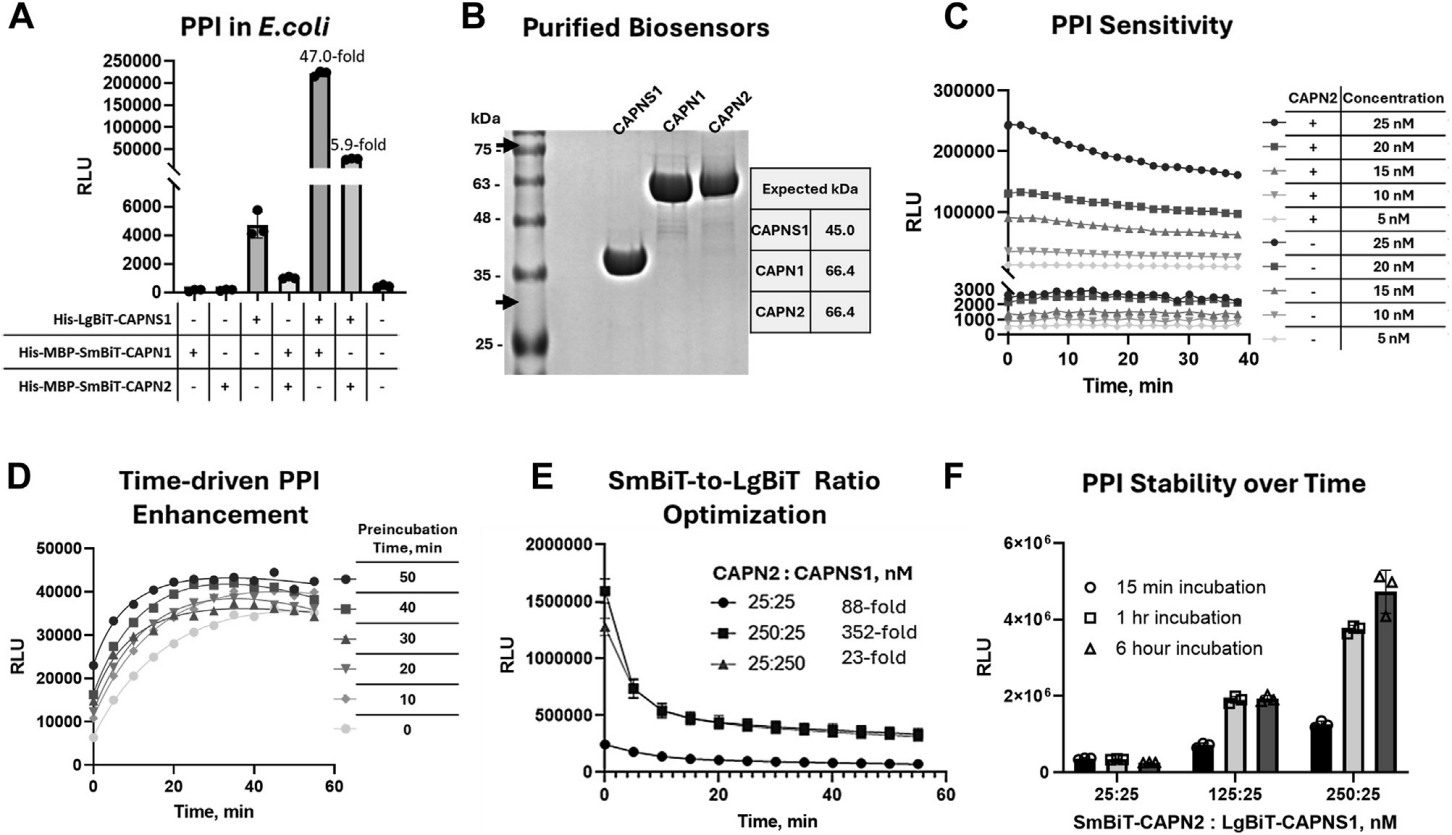
**Optimization of bacterially expressed affinity-purified CAPNS1–CAPN1/2 biosensors.** The assays were conducted in the presence of 5 mM Mg^2+^ and in the absence of Ca^2+^. Individually expressed biosensors were preincubated for at least 60 min before the addition of furimazine substrate, and the resulting luminescent signal was read 15 min after the addition of the substrate, unless otherwise indicated. *A,* luciferase assays of approximately equimolar amounts of recombinant CAPNS1–CAPN1/2 biosensor components from bacterial lysates. The fold changes indicate the signal increase over the negative control, which was His_10_-LgBiT-CAPNS1 alone. *B,* Coomassie blue–stained SDS-PAGE of 20 μg of each affinity–purified biosensor component. The observed purities of His_10_-LgBiT-CAPNS1, His_6_-MBP-SmBiT-CAPN1, and His_6_-MBP-SmBiT-CAPN2 components are 75.7%, 80.3%, and 78.1%, respectively. *Black arrows* indicate the sizes of full-length CAPNS1 and CAPN1/2 to 28 kDa and 80 kDa, respectively. *C,* luciferase assays of the purified recombinant CAPNS1–CAPN2 biosensor with equimolar amounts of the two components (+) or the His_10_-LgBiT-CAPNS1 component alone (−) ranging from 5 to 25 nM assayed over 40 min. *D,* CAPNS1–CAPN2 biosensor components were preincubated for the indicated times before addition of furimazine substrate and then assayed over 60 min. *E,* luciferase assays of CAPNS1–CAPN2 biosensor components combined at the indicated molar ratios and assayed over 60 min. *F,* luciferase assays of CAPNS1–CAPN2 biosensor components at the indicated molar concentration ratios after preincubation for the indicated times. In (*A*) and (*F*), each data point represents a measurement of an independent reaction mixture.

The optimal CAPN1/2 PEF(L) and CAPNS1 PEF(S) biosensor components were cloned into bacterial expression plasmids, and the corresponding recombinant proteins (Fig. 2*C*) were expressed in BL21 RIPL *Escherichia coli* cells (Fig. S1). Using combinations of crude bacterial lysates with approximately equimolar concentrations of the biosensor components, the CAPN1-CAPNS1 and CAPN2-CAPNS1 biosensors produced 47-fold and 5.9-fold stronger signals than the LgBiT-CAPNS1–alone control (Fig. 3*A*). The weaker apparent PPI affinity of the CAPN2–CAPNS1 relative to the CAPN1–CAPNS1 biosensor was consistent with observations made by probing synthetic peptide arrays, which are useful tools for probing interaction motifs and relative strength of precise PPIs (26, 27). Arrays containing overlapping 25mers spanning the CAPN1 or CAPN2 PEF domains were incubated with lysates of HEK293T cells expressing a Myc-epitope tagged CAPNS1 protein. Immunoblotting detection with anti-Myc antibody yielded strongest signals on peptides corresponding to the fifth EF hand of the CAPN1 PEF domain (Fig. S2).

The bacterially expressed biosensor constructs were next affinity-purified on nickel–nitrilotriacetic acid columns, yielding over 50 mg of each highly purified protein (His_10_-LgBiT-CAPNS1, His_6_-MBP-SmBiT-CAPN1, and His_6_-MBP-SmBiT-CAPN2) with the expected molecular weights (Fig. 3*B*). Since CAPN1 and CAPN2 are paralogs with 53% amino acid sequence identity and 78% amino acid sequence similarity in the PEF domain, further optimizations were performed on the CAPN2–CAPNS1 biosensor alone. To note, sequence identity between all three CAPNS1, CAPN1, and CAPN2 is 31%, and sequence similarity is 64% (shown in sequence (28) alignment in the Table S7).

The sensitivity of the purified CAPN2–CAPNS1 biosensor assay was explored through serial dilution. Strong luminescence signals were achieved with as low as 5 nM equimolar concentrations of each biosensor component (Fig. 3*C*) with the signal over 20-fold higher in the CAPN2–CAPNS1 biosensor mix than in the LgBiT-CAPNS1–alone control at 5 nM concentration at 30 min. The assay was further optimized by testing protein preincubation times (Fig. 3*D*) and the ratio of the two components (Fig. 3*E*). Changing the ratio from 1:1 to 10:1 in either direction (LgBiT:SmBiT or SmBiT:LgBiT) increased the signal identically, but the signal-to-noise ratio was improved with 10:1 SmBiT:LgBiT and made worse with 10:1 LgBiT:SmBiT. This is consistent with a measurable amount of background signal originating from LgBiT alone. The biosensor maintained stable signal strength after 6 h of incubation at room temperature, ensuring convenient utility in applications that might require prolonged incubation times (Fig. 3*F*).

Separately, the PPIs of the biosensor constructs were visualized in the native state by size-exclusion chromatography (Fig. S4*A*), blue native PAGE (Fig. S4*B*), and blue native PAGE followed by immunoblot (Fig. S4, *C* and *D*), which confirmed the presence of monomers and dimers for the recombinant CAPN1, CAPN2, and CAPNS1 proteins. Most notably, LgBiT-CAPNS1 monomers, abundantly present in CAPNS1 preparation, are segregated to the dimer protein bands once incubated with either SmBiT-CAPN1 or SmBiT-CAPN2 (Fig. S4, *B* and *C*), which accounts for the LgBiT-SmBiT reconstitution and thus luminescence. Such observation was abolished when LgBiT-CAPNS1 was instead incubated with a substoichiometric concentration of SmBiT-CAPN1 or SmBiT-CAPN2.

### Validation of the CAPNS1–CAPN1/2 PPI biosensors

**Figure 4:**
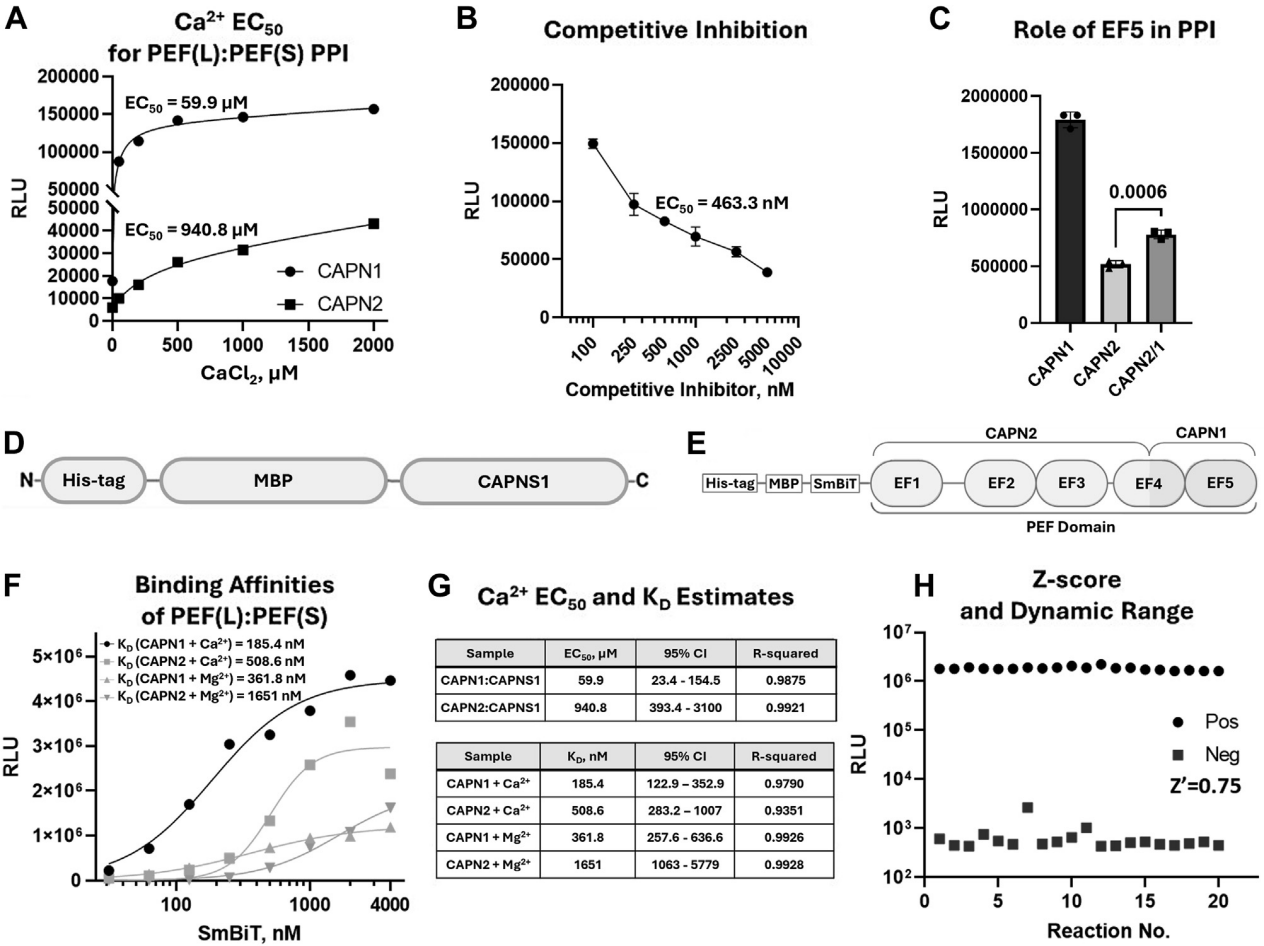
**Characterization and performance analysis of the CAPNS1–CAPN1/2 PEF(S)–PEF(L) PPI biosensors: Ca^2+^ responsiveness, reproducibility, and inhibitor effects.***A,* Ca^2+^ dose–response analysis of CAPNS1–CAPN1/2 biosensors in the presence of 5 mM Mg^2+^ reveals Ca^2+^-enhanced PPI; EC_50_ = 59.9 μM and EC_50_ = 940.8 μM, respectively. *B,* competitive inhibition of the CAPNS1–CAPN2 biosensor (25 nM CAPNS1 and 250 nM CAPN2) with increasing concentrations of “LgBiT-less” CAPNS1, shown in (*D*); IC_50_ = 463.3 nM. *C,* substituting the C-terminal 42 amino acids of CAPN2 with the homologous sequences from CAPN1 (chimeric protein CAPN2/1 shown in (*E*) increased the biosensor PPI by 50.7% [one-way ANOVA *F*(2, 6) = 555.0, *p* < 0.0006]). *F,* addition of increasing amounts of the SmBiT-CAPN1/2 biosensor component to a fixed concentration of LgBiT-CAPNS1 (100 nM) in the presence of 5 mM of either Ca^2+^ or Mg^2+^ produced first order–like sigmoidal binding kinetics and the estimated *K*_*D*_ values. *G,* quantification of the Ca^2+^ binding EC_50_ values for the calpain-1 and calpain-2 PPI (*upper*; data from *A*); and quantification of the calpain dissociation constants (*K*_*D*_) based on the biosensor signal in the presence of either 5 mM Ca^2+^ or Mg^2+^ (*lower*; data from *F*). *H,* the optimized CAPNS1–CAPN2 biosensor (250 nM SmBiT-CAPN2, 25 nM LgBiT-CAPNS1) is highly reproducible with a *Z*-score of 0.75 and a large dynamic range. Each data point represents a measurement of an independent reaction mixture. PEF, penta-EF-hand domain; PPI, protein–protein interaction.

**Figure 5:**
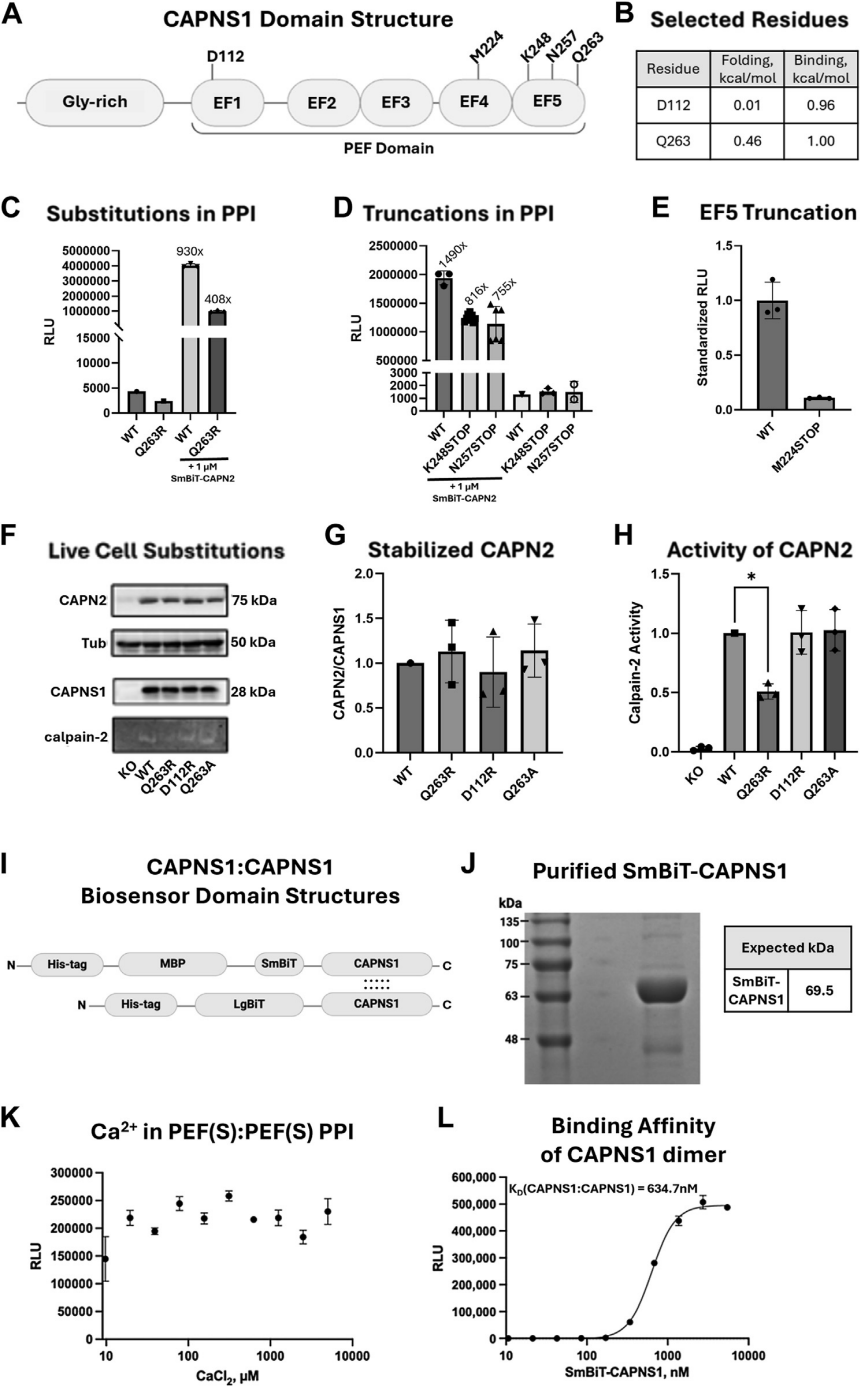
**Validation of *in silico* predictions using purified biosensor proteins and mammalian in cell stabilization and proteolytic activity analyses.***A,* domain map of CAPNS1 with mutation sites indicated relative to individual EF motifs of the PEF domain. *B, in silico*–predicted effects of mutating the indicated residues on monomer folding and heterodimer binding energies. *C,* CAPNS1–APN2 biosensor changes with the CAPNS1 Q263R point mutation, measured in bacterial lysates. 1 μM SmBiT-CAPN2 was used for the PPI measurement in a buffer containing 5 mM Ca^2+^. Luminescence produced from the LgBiT-CAPNS1 alone, either WT or mutant, has been measured to quantify relative protein expression. Data are presented as RLUs and as RLU fold changes over those of the respective LgBiT-alone controls. *D,* CAPNS1–CAPN2 biosensor changes with the indicated truncations, measured in bacterial lysates, as described for *C*. *E,* CAPNS1–CAPN2 biosensor changes with the complete truncation of EF5, measured with purified proteins at 25 nM for each component in the presence of 5 mM Ca^2+^. *F,* exogenous expression of the indicated recombinant CAPNS1 proteins in *CAPNS1* CRISPR KO MDA-MB-231 cells. Immunoblotting of cell lysates for CAPN2, tubulin, and CAPNS1 in the upper three panels; and casein zymography analysis of calpain-2 proteolytic activity in the *bottom panel*. *G,* densiometric quantification of recombinant CAPNS1-induced rescue of CAPN2 expression, standardized to CAPNS1 expression. No statistically significant difference was found (one-way ANOVA *F*(3, 6) = 0.3113, *p* = 0.817). *H,* densiometric quantification of recombinant CAPNS1-induced rescue of calpain-2 casein proteolytic activity, standardized to CAPN2 expression. Q263R produced a statistically significant deviation of calpain-2 activity (51.0 ± 6.4% of WT, one-way ANOVA *F*(3, 6) = 7.813, *p* = 0.031). *I,* domain diagrams of biosensor constructs, expressed in bacterial cells, for analysis of the PEF(s):PEF(S) homodimer PPI. Constructs contain oligo histidine (His-tag), maltose-binding protein (MBP), LgBiT and SmBiT of the split-Nanoluciferase, and amino acids 60 to 268 of CAPNS1. *J,* SDS-PAGE of 20 μg of affinity-purified His_6_-MBP-SmBiT-CAPNS1 biosensor stained by Coomassie blue. His_6_-MBP-SmBiT-CAPNS1 had an observed purity of 79%. The expected atomic mass of His_6_-MBP-SmBiT-CAPNS1 is 69.5 kDa. *K,* Ca^2+^ dose–response analysis of SmBiT-CAPNS1 and LgBiT CAPNS1 biosensors. Linear regression slope is not statistically significantly different from zero (Wald test, *F*(1, 28) = 0.1779, *p* = 0.6764), suggesting no Ca^2+^ effect. *L,* first order–like sigmoidal binding kinetics and estimated *K*_*D*_ value obtained from measuring luminescence of 100 nM LgBiT-CAPNS1 with an increasing concentration of SmBiT-CAPNS1 in the presence of 5 mM Ca_2_^2+^. Observed PEF(S):PEF(S) PPI *K*_*D*_ is 634.7 nM (95% CI 606.2–665.1 nM). *C*–*E,* each data point represents a measurement of an independent reaction mixture. *G* and *H,* each data point represents quantification of an independent immunoblot or a zymogram. PEF, penta-EF-hand domain; PPI, protein–protein interaction; RLU, relative light unit.

The calpain-1 or calpain-2 enzymes have a total of 10 binding sites for Ca^2+^, including one in each of the PC1 and PC2 subdomains of the catalytic core and four in each of the PEF(S) and PEF(L) domains (2). Ca^2+^ binding to the two sites in PC1 and PC2 subdomains correlates with conformational changes in the catalytic core, which align the three amino acids of the catalytic triad (Fig. 1); and this is thought to be responsible for activation (18, 20, 21). Ca^2+^ binding at the other eight sites in the PEF domains may promote the PEF(S)–PEF(L) PPI or other intramolecular interactions of these PEF domains with other regions of the catalytic subunit, including the N-terminal anchor helix (2). As such, the CAPNS1–CAPN1/2 biosensors were shown to be responsive to Ca^2+^ concentrations (Fig. 4, *A* and *G*); which enabled EC_50_ estimates for the optimal Ca^2+^ concentrations for the PEF(S)–PEF(L) PPI of 59.9 and 940.8 μM, for calpain-1 and calpain-2, respectively. These values are consistent with existing knowledge of the optimal Ca^2+^ concentrations for proteolytic activation of calpain-1 and calpain-2 of ∼42 μM and >1 mM, respectively (18, 29). The calpain regulatory subunit homodimer has eight available Ca^2+^-binding sites, similarly to the heterodimer. And yet, interestingly, PEF(S) homodimerization was not enhanced with increasing calcium concentration (Fig. 5*K*).

The optimized biosensors were next used to estimate the dissociation constants of calpain-1 and calpain-2 heterodimers by performing CAPNS1–CAPN1/2 biosensor assays with a constant concentration of the His_10_-LgBiT-CAPNS1 component and increasing concentrations of His_6_-MBP-SmBiT-CAPN1/2 (Fig. 4*F*) or His_6_-MBP-SmBiT-CAPNS1 (Fig. 5*L*) components. Assuming first-order kinetics, the inflection values in the resulting plots are representative of the *K*_*D*_ of the reactions. The *K*_*D*_ values for CAPN1:CAPNS1 or CAPN2:CAPNS1 were 185.4 or 508.6 nM, respectively, in the presence of 5 mM Ca^2+^; and 361.8 or 1651 nM, respectively, in the presence of Mg^2+^ in place of Ca^2+^ (Fig. 4, *F* and *G*). These findings provide evidence for a higher affinity of the PEF(S)–PEF(L) PPI in calpain-1 relative to calpain-2; and a preference for Ca^2+^ for efficient dimerization relative to the similar divalent ion, Mg^2+^. These observations also suggest a contributing role for Ca^2+^ binding in the PEF domains toward proteolytic activation through strengthening the heterodimer PPI. Yet, Ca^2+^ had no effect on CAPNS1 homodimerization (Fig. 5*K*). The *K*_*D*_ value for the CAPNS1:CAPNS1 homodimer was 634.7 nM (Fig. 5*L*). This indicates that, in the presence of Ca^2+^, the PEF(S):PEF(S) homodimer has weaker affinity than both the calpain-1 and calpain-2 PEF(S):PEF(L) heterodimers.

Next, we expressed a chimeric CAPN2/1 biosensor component that was based on His_6_-MBP-SmBiT-CAPN2, but with the CAPN2 C-terminal 42 residues (the C-terminal helix–loop–helix, containing EF5 and a portion of EF4) replaced with the homologous CAPN1 sequences (amino acids 672–714). The resulting chimeric CAPN2/1 gained a significantly stronger affinity for CAPNS1 than CAPN2 (Fig. 4*C*), indicating that the biosensor is responsive to structural changes, the EF5 motif plays an important role in the PPI, and this EF5 motif–mediated interaction contributes to the higher affinity of CAPN1 with CAPNS1. We also showed that the CAPNS1–CAPN2 PPI could be attenuated in a dose-dependent manner with a competitive inhibitor consisting of the His_6_-MBP-CAPNS1 protein without the LgBiT (Fig. 4*B*).

In summary, we have made highly reproducible calpain-1 and calpain-2 PPI biosensors using the split Nanoluciferase system. These biosensors display highly reproducible controls and large dynamic ranges with *Z*-factors of 0.75 (Figs. 4*H*; S3) (30).

### Defining critical residues mediating the calpain-2 heterodimer PPI *via in silico* modeling

Using the 3D structure of the calpain-2 heterodimer, we carried out two types of calculations for residues at the CAPNS1–CAPN2 interface: (a) predicting the effect of individual mutations (alanine substitutions) on the stability of those mutated unbound subunits and (b) predicting the effect of these individual mutations on the binding free energy of the CAPNS1–CAPN2 heterodimer. The first type of calculations were done to avoid mutations that make the unbound mutant subunit unstable and would thus indirectly affect the heterodimerization. The second type of calculations were done to assess the effect of mutations on the affinity of the mutated subunit for its partner in the heterodimer, while still being stable and properly folded in the unbound state. Predictions were made with the most popular and accurate methods (list is provided in the *Experimental procedures* section), and then the results averaged to result in average DDG_folding_ and DDG_binding_. The results of individual predictors are shown in Tables S1 and S2. Following this approach, mutations with an absolute value of average change of folding free energy greater than 0.5 kcal/mol (*i.e.*, average |ΔΔG_folding_ >0.5 kcal/mol) were excluded from further consideration, as these would likely result in destabilized proteins. The rankings of the remaining mutations based on ΔΔG_binding_ from each method (list is provided in the *Experimental procedures* section) were then averaged to obtain a consensus ranking. The top 20 most disruptive dimerization mutations are shown in Table 1 along with the corresponding average DDG_binding_ and DDG_folding_. The ΔΔG_binding_ average values of these selected 20 mutations do not always decrease in order from top to bottom because the ranking is based on the consensus of rankings obtained from each prediction method, not the average ΔΔG_binding_ values.

**Table 1:** Consensus ranking of top 20 mutations at interfacial residues of human calpain-2

Final rank	Protein	Position	Residue	Average |ΔΔG_folding_| (kcal/mol)	Average ΔΔG_binding_ (kcal/mol)
1	CAPN2	417	R	0.32	1.15
2	CAPNS1	154	D	0.27	0.97
3	CAPNS1	111	D	0.02	1.02
4	CAPNS1	112	D	0.01	0.96
5	CAPNS1	263	Q	0.46	1.00
6	CAPNS1	118	T	0.26	0.96
7	CAPN2	690	D	0.08	0.68
8	CAPN2	653	D	0.48	0.84
9	CAPN2	7	K	0.03	0.73
10	CAPNS1	119	E	0.27	0.84
11	CAPN2	367	R	0.48	0.75
12	CAPNS1	265	T	0.32	0.80
13	CAPN2	418	R	0.45	0.80
14	CAPN2	420	R	0.27	0.74
15	CAPN2	582	D	0.15	0.76
16	CAPNS1	155	T	0.33	0.73
17	CAPN2	575	E	0.43	0.79
18	CAPNS1	163	E	0.26	0.76
19	CAPN2	661	N	0.47	0.69
20	CAPN2	494	D	0.36	0.60

Following this *in silico* modeling, we performed structure–function analyses to validate the roles of selected residues in CAPNS1 (Fig. 5, *A* and *B*). Introduction of a Q263R point mutation into the CAPNS1 biosensor component reduced its binding affinity with CAPN2 by 56% (Fig. 5*C*). We further explored this Q263R mutation, as well as D112R and Q263A point mutations, in a mammalian cell model. A *CAPNS1* CRISPR KO MDA-MB-231 human triple-negative breast cancer cell line (null for CAPNS1 expression) (31) was transduced with retroviruses encoding Q263R, D112R, and Q263A mutants, or WT recombinant CAPNS1 proteins (Fig. 5*F*). As loss of CAPNS1 results in destabilization of endogenous CAPN2 and CAPN1, this model allowed us to measure how well these mutant CAPNS1 constructs rescued the expression of the CAPN2 subunit (Fig. 5, *F* and *G*) and the proteolytic activity of the rescued calpain-2 heterodimer (Fig. 5, *F* and *H*).

Ectopic expression of CAPNS1, WT, or Q263R, or D112R, or Q263A, rescued the expression of CAPN2, suggesting that these mutant calpains were able to bind and stabilize CAPN2. There was no statistically significant difference in the apparent expression of the stabilized CAPN2, standardized to CAPNS1 (one-way ANOVA *F*(3, 6) = 0.3113, *p* = 0.817). Ectopically expressed CAPNS1 constructs were also able to rescue the proteolytic activity of calpain-2 enzyme, as seen on the zymogram (Fig. 5, *F* and *H*). However, CAPNS1 Q263R statistically significantly attenuated the calpain-2 activity, down to 51.0 ± 6.4% of the WT (one-way ANOVA *F*(3, 6) = 7.813, *p* = 0.031). This functional analysis was largely consistent with the *in silico* predictions and validated the essential nature of specific residues. The Q263R result also aligns with the AlphaMissense prediction that the Q263R is likely pathogenic (0.75 cutoff) (32).

The aforementioned data provide evidence that the C terminus of CAPNS1 could be a critical region mediating calpain-2 heterodimerization. To explore this, we introduced stop codons at positions N257, K248, and M224 into the CAPNS1 biosensor component and tested their effects on the CAPNS1–CAPN2 biosensor. Truncations at N257 and K248, which removed portions of the EF5 motif, reduced the PPI strength to 58.4% and 64.1%, respectively (Fig. 5*D*); and a stop codon at M224, which completely removed EF5, reduced the PPI down to 11.0% of WT (Fig. 5*E*).

## Discussion

Proteases, the largest enzyme gene family in vertebrates, comprise over 600 genes in the human genome (33). Their inhibition, pharmaceutical or genetic, has far-reaching implications, ranging from altering cellular protein homeostasis (34) in an individual cell to disrupting clotting cascades (35) organism wide. Inhibition of calpains is of a particular interest to us due to this protein family's involvement in cancer, fibrosis, neurodegenerative, and cardiovascular disease (reviewed in Ref. (9)). There have been many approaches tried and tested in efforts to bring calpain inhibition to the clinic. Most often, small-molecule and peptidomimetic active site–directed approaches are used (36). However, these agents are promiscuous in an *in vivo* setting, which has limited their clinical suitability. Another approach is to leverage the highly specific endogenous calpain-1 and calpain-2 inhibitor calpastatin (CAST). Specific and potent inhibitors of these calpain isoforms have been developed based on CAST primary sequence (37). However, they tend to be poorly membrane permeable and are rapidly cleared through the kidneys *in vivo*. While it is possible to derivatize CAST to make it blood–brain barrier permeable (8), no such drug has entered clinical trials yet, underscoring the challenge of translating a good *in vitro* pharmacophore into a viable drug.

The development of selective and effective inhibitors of calpain proteases remains an elusive challenge, both in the clinical setting (with no currently available approved inhibitors); and to a lesser extent, in the laboratory, although there are several effective, but nonspecific, inhibitors that are widely used in research applications (reviewed in the study by Ono *et al.*, 2016) (9). This challenge can be largely attributed to two limitations. First, it is difficult to specifically measure calpain activity *in vivo*; which makes inhibitor assay development challenging. Second, and related to the first limitation, the homology between the active site of calpain isoforms and those of approximately 600 (38) other human proteases makes it difficult to develop a selective active site–directed calpain inhibitor. To circumvent these limitations in the case of classical calpain-1 and calpain-2 isoforms, we suggest targeting the obligatory PPI of their catalytic and regulatory subunits instead of their active sites. In principle, this offers an opportunity for greater selectivity since the PEF domains that mediate these PPIs are infrequent structures in the human proteome. Other than the nine classical PEF domain–containing calpain isoforms (1, 2, 3, 8, 9, 11, 12, 13, and 14), the PEF domain family contains only five other proteins: ALG-2, ALG-2-like, peflin, grancalcin, and sorcin (17), which limits the scope of potential allosteric off-target effects. As a first step in pursuing this targeting strategy, we have developed split-Nanoluciferase PPI biosensors that precisely measure the PEF–PEF PPIs of calpain-1 and calpain-2. In creating the biosensors, we balanced the biochemical activity of the fusion proteins, their stability, and their homology. The optimal orientations were achieved by fusing LgBiT or SmBiT to the N termini of the PEF domains of CAPN1, CAPN2, and CAPNS1. This avoided undesirable misfolding of the PEF domains when their C termini were perturbed (Fig. 2*B*). Using these fusion protein split-Nanoluciferase calpain PPI biosensors, we have measured the affinity of PEF(L)–PEF(S) associations for both calpain-1 and calpain-2 and showed that this association is significantly stronger in calpain-1 than in calpain-2. This is consistent with peptide array binding studies showing stronger binding of CAPNS1 to CAPN1 peptides corresponding to the C-terminal EF5 motif, compared with the homologous CAPN2 peptides (Fig. S2). It should also be noted that this higher affinity for the PEF(L)–PEF(S) PPI of CAPN1–CAPNS1 correlates with a lower Ca^2+^ concentration required for calpain-1 protease activation relative to that of calpain-2 (18).

Previous structural studies have provided evidence that Ca^2+^ binding at sites in each of the PC1 and PC2 domains of calpain-2 correlates with alignment of the catalytic triad residues (C105, H262, and N286); and therefore, is likely to play an essential role in catalytic activation (18, 20, 21). Here, we show novel evidence that Ca^2+^ binding at the four sites in each of the PEF domains strengthens the association between the catalytic and regulatory subunit. Taken in conjunction with the knowledge that expression of CAPNS1 is necessary for the stability and protease activity of calpain-1 and calpain-2 (11, 12), our new observations provide indirect evidence that Ca^2+^ binding to EF hand motifs in the PEF domains may also contribute to catalytic activation through tightening the PPI. However, *in vitro* experiments have shown that the core domain of CAPN1 (without the PEF domain) has proteolytic activity, so this argues that PEF–PEF interactions are not essential for calpain activity (18). Therefore, while we cannot conclude that the PEF(L)–PEF(S) PPI is required for protease activity, our observations suggest it may still contribute to that activation. Furthermore, given the absence of detectable CAPN1 or CAPN2 subunits in *CAPNS1* KO cells (6, 7), it seems likely that stability of these catalytic subunits of calpain-1 and calpain-2 are dependent on the PPI with CAPNS1. It follows that strategies capable of disrupting these PPIs would downregulate the steady state levels of these classical calpains.

There is evidence that Mg^2+^ can partially activate calpain, and Mg^2+^ can be outcompeted by Ca^2+^ to further increase catalytic activity (39). Our CAPNS1–CAPN1/2 biosensor PPI binding curves and the resulting dissociation constants obtained with increasing concentrations of Ca^2+^, in the presence of 5 mM Mg^2+^, suggest that divalent ion binding sites in the PEF domains of calpain-1 and calpain-2 may be fully occupied with Mg^2+^, which is available at approximately 1 to 20 mM concentration within different compartments of a cells (40). The resting Ca^2+^ concentration in a cell is approximately 100 nM (41), which is insufficient for activation of endogenous calpain-1 or calpain-2. This suggests that these enzymes must be activated by Ca^2+^ influx from the extracellular space, where Ca^2+^ concentration is 1.1 to 1.4 mM (42), or by Ca^2+^ release from intracellular stores, such as the endoplasmic reticulum (42). Similarly to enzymatic activation of calpain-1 and calpain-2 proteases, our PPI biosensors were activated to half-maximum with 59.9 μM and 940.8 μM Ca^2+^, respectively. On the contrary, the CAPNS1 homodimer PPI does not appear to be Ca^2+^ dependent, which is in line with previous evidence that the Ca^2+^-bound CAPNS1 homodimer has no remarkable structural deviations compared with the Ca^2+^-free heterodimer (21), suggesting that Ca^2+^ binding does not significantly alter homodimer formation, whereas heterodimer PEF–PEF interactions are enhanced by Ca^2+^. Together, the data suggest that “tightening” of the heterodimer PPI through PEF–PEF interactions may play a role in enzyme activation, but a putative homodimer PPI would be unaffected by fluctuations in Ca^2+^.

While the split-Nanoluciferase calpain biosensor system has proven very sensitive in measuring changes in PEF:PEF PPI, it is important to note that in a full-size endogenous calpain, the interaction between CAPN1 or CAPN2 with CAPNS1 is not limited to the hydrophobic PEF domain interface. Residue D112 of CAPNS1, for example, which produces an electrostatic interaction with positively charged residues in the PC1 domain (shown in Fig. 1*A*), is predicted to have a significant effect on calpain dimerization free energy (Table 1). Yet, even a charge reversing D112R mutation did not result in significant effects on calpain stability or activity (Fig. 5, *G* and *H*). However, a point mutation in Q263 of the CAPNS1 EF5 did weaken the PPI (Fig. 5*C*), and C-terminal truncations of EF5 abolished the PEF:PEF PPI (Fig. 5, *D* and *E*). This underscores that the hydrophobic EF5:EF5 interaction is the main source of affinity between subunits and is therefore an ideal target for allosteric PPI inhibitions.

Similar to the *in vitro* protease activity effects of Mg^2+^ and Ca^2+^ described by Gaitanaki *et al.* (39), we found that Ca^2+^ addition in the presence of 5 mM Mg^2+^ promoted stronger PPI affinities with our biosensors. The Ca^2+^ dose–response analysis of the biosensors suggests a significant contribution of the PEF–PEF PPI to the activation of calpain-1/2 enzymatic activity. Interestingly, some previous studies suggested that Ca^2+^ binding may induce calpain subunit dissociation or aggregation under specific *in vitro* conditions (22, 43). However, contradictory findings indicate that Ca^2+^ stabilizes dimerization rather than promoting dissociation, depending on ionic strength and cellular context (44). By utilizing a split-Nanoluciferase system to isolate the PEF(S)–PEF(L) interaction, we provided real-time quantitative evidence supporting the latter model, demonstrating that Ca^2+^ enhances heterodimerization affinity for both calpain-1 and calpain-2. These findings suggest that earlier reports of dissociation may reflect secondary effects from other domains rather than an intrinsic property of the PEF–PEF interaction.

Competitive inhibition of the CAPNS1–CAPN2 biosensor using untagged CAPNS1 provides evidence that allosteric inhibition of calpain PEF–PEF is feasible. Crystal structures of calpain-2 have revealed evidence for EF1/EF2 and EF3/EF4 intrasubunit interactions, leaving the “unpaired” EF5 motifs to engage in intersubunit interactions that have been proposed to play prominent roles in heterodimerization (45). This argues that targeting these EF5/EF5 intersubunit interactions could interfere with heterodimerization. This was supported by our observations that deletion of part or all EF5 in the CAPNS1 biosensor component partially or completely abolished the PPI, respectively. Stable expression of CAPNS1 mutants lacking these C-terminal sequences was not possible to achieve in mammalian cells, which prevented us from testing their ability to restore stable expression of endogenous CAPN1 and CAPN2 or evaluate calpain-1 or calpain-2 proteolytic activity *in vivo* using the *CAPNS1* KO MDA-MB-231 breast cancer cell model. However, we were able to achieve stable mammalian cell expression of several CAPNS1 mutants with single amino acid substitutions, and these achieved stabilization of endogenous CAPN1 and CAPN2, as well as detectable levels of calpain-2 activity in *CAPNS1* KO MDA-MB-231 cell lysates (Fig. 5, F and G,H). The most deleterious point mutation evaluated was the Q263R mutation, which corresponds to a residue in the CAPNS1 EF5 motif; and interestingly, this was one of the highest ranked residues in our *in silico* modeling analysis (Table 1).

Our conclusion that the C-terminal regions of CAPNS1 (EF5 and part of EF4) are the most important for heterodimerization is perhaps not surprising in the light of the crystal structures of calpain-2. Yet, the interfacial area of the heterodimer is much larger, and currently, there is little insight about key residues or “hot spots” that might make strong contributions to heterodimerization. The identification of PPI hot spots is critical for the development of approaches aimed at modulating heterodimerization. Thus, an important contribution of this study is the identification of several residues at the interface of the heterodimer, which should be considered as targets for allosteric inhibition of calpain-1 and calpain-2.

A limitation of our work is that the PEF–PEF biosensors lack the context of the rest of the catalytic subunit. The crystal structure of calpain-2 reveals that the PC1 domain has a prominent contact surface with PEF(S) in calpain-2; and the N-terminal anchor helix, which is in contact with PEF(S) in some published crystal structures is also suspected to play a role in calpain-2 activation.

Separately, CAPNS1 is known to be able to form homodimers in *in vitro* model systems (45). The PEF(L) domains of the calpain-1 and calpain-2 catalytic subunits are significantly similar to the PEF(S) domain of the calpain regulatory subunit CAPNS1, in sequence and in structure (46), which suggests that the calpain regulatory subunit may be similarly capable of forming PEF:PEF homodimers in an endogenous setting through a hydrophobic association of two-fifth EF-hand motifs, like the association that stabilizes calpain-1 and calpain-2 heterodimers. There is no *in vivo* proof of such a dimer, but protein crystallization studies have revealed that CAPNS1 can indeed be crystalized in the form of a PEF(S):PEF(S) homodimer of two CAPNS1 subunits (1, 21), which shows that such an interaction *in vivo* is at least plausible. The ability of CAPNS1 to form a PEF(S):PEF(S) homodimer was confirmed with the split Nanoluciferase PPI biosensor system. Simple first-order–like kinetics were observed, and the *K*_*D*_ was estimated at 634.7 nM (95% confidence interval: 606.2–665.1 nM). This PEF(S):PEF(S) homodimer was not responsive to Ca^2+^ (*p* = 0.68), in contrast to the calpain-1 and calpain-2 PEF(L)–PEF(S) heterodimers. This is in agreement with a previous analysis of a CAPNS1 homodimer crystal, which revealed that the PEF(S) domain of CAPNS1 undergoes minimal conformation changes upon Ca^2+^ binding (21, 45).

It should also be mentioned that the reported changes of the binding free energy in Table 1 are possibly underestimated. This stems from a recent study showing that computational methods, when benchmarked against experimental data, underestimated the ΔΔG_binding_ by a factor of approximately 2 (25). These limitations, together with experimental validation that single mutations can only partially disrupt the PPI, suggest that larger portions of the PPI interface would likely need to be targeted to effectively prevent heterodimerization.

To summarize, we have measured calpain PEF–PEF PPIs for calpain-1 and calpain-2, which has never been done before; we have demonstrated that it can be disrupted to reduce calpain activity; and we have predicted hot spot residues for disruption of the CAPN2–CAPNS1 PPI. Together, these data support the strategy of allosteric inhibition of calpain-1 and calpain-2 through interference with their obligatory heterodimer structures.

## Experimental procedures

### Plasmid construction and mutagenesis

Sequences encoding human CAPNS1 (amino acids 60–268), CAPN1 (amino acids 538–714), and CAPN2 (amino acids 524–700) were cloned from complementary DNA (cDNA) generated with total RNA isolated from the human triple-negative breast cancer cell line MDA-MB-231 using the SuperScript IV CellsDirect cDNA synthesis kit according to the manufacturer's instructions (ThermoFisher; catalog no.: 11750150). The respective coding sequences were PCR amplified with KOD Hot Start DNA Polymerase (MilliporeSigma; catalog no.: 71086) from the total cDNA using the oligonucleotide primers shown below, digested with the indicated restriction endonucleases, and cloned between those sites in the NanoBiT Protein:Protein Interaction System mammalian expression vectors pBiT1.1-N (TK/LgBiT) and pBiT2.1-N (TK/SmBiT) (Promega; catalog no.: N2014) to achieve in-frame fusions with sequences encoding LgBiT or SmBiT. For brevity, primer sequences for the unsuccessful combinations are not shown.

**Table:** 

XhoI-CAPNS1-Fw NheI-CAPNS1-Rv	CGCCGCTCGAGCCGCATCCTAGGCGGAGTCATCAG CTACCGCTAGCTTAGGAATACATAGTCAGCTGCAGCCACTC
XhoI-CAPN1-Fw NheI-CAPN1-Rv	CGCCGCTCGAGCCTCTCAGAAGAGGAGATTGACGAGAACT CTACCGCTAGCTTATGCAAACATGGTCAGCTGCAACCACTT
XhoI-CAPN2-Fw NheI-CAPN2-Rv	CGCCGCTCGAGCGACATCAGCGAGGATGACATTGA CTACCGCTAGCTTAAAGTACTGAGAAACAGAGCCA

The optimal CAPNS1 PEF(S) biosensor component was PCR amplified and cloned into pET16b (MilliporeSigma; catalog no.: 69662) between NdeI and BamHI; and the optimal CAPN1 or CAPN2 PEF(L) biosensor components were PCR amplified and cloned between BamHI and NdeI, or NdeI and SalI, respectively, in a modified pET16b plasmid (HT29 was a gift from Zongchao Jia, Queen's University) (47) using the primers shown below.

**Table:** 

CAPNS1	pET16b NdeI–BamHI	Fw: AAAGCCCATATGGTCTTCACACTCGAAGATTT Rv: AGATCTGGATCCTTAGGAATACATAGTCAGCT
CAPN1	pHT29 BamHI–NdeI	Fw: GAAGGTGGATCCATGGTGACCGGCTACCGGCTGTTCG Rv: AACTTCCATATGTTATGCAAACATGGTCAGCTGCAAC
CAPN2	pHT29 NdeI–SalI	Fw: GAAGGTCATATGATGGTGACCGGCTACCGGCTGTTCG Rv: ACCTTCGTCGACTTAAAGTACTGAGAAACAGAGCCAA

The complete DNA sequences of the SmBiT and LgBiT CAPN1/2/S1 biosensor constructs are shown in Tables S3–S6.

A chimeric calpain biosensor was made by PCR amplifying the CAPN1 C terminus (amino acids 672–714) using the primers shown below; and cloning this CAPN1 C terminus into pHT29_SmBiT_CAPN2 between the ClaI sites to replace the homologous CAPN2 C-terminal sequences.

**Table:** 

Fw	CTGGCGATCGATTTTGACAATTTCGTTTGCTGCCTGG
Rv	CTTATCATCGATAAGCTTTAATGCGGTAGTTTATCAC

Q263, D112, M224, K248, and N257 mutations were introduced into pET16b_LgBiT_CAPNS1 or pMSCV_CAPNS1 using QuikChange II XL Site-Directed Mutagenesis Kit (Agilent; catalog no.: 200517) in accordance with its standard protocol using the primers shown below.

**Table:** 

D112R	Fw: CAGCTGGCTGGAGATCGCATGGAGGTCAGCGCC Rv: GGCGCTGACCTCCATGCGATCTCCAGCCAGCTG
Q263A	Fw: ATCCAGGAGTGGCTGGCCCTGACTATGTATTCC Rv: GGAATACATAGTCAGGGCCAGCCACTCCTGGAT
Q263R	Fw: ATCCAGGAGTGGCTGCGCCTGACTATGTATTCC Rv: GGAATACATAGTCAGGCGCAGCCACTCCTGGAT
N257STOP	Fw: AGATGGCACTGGACAAATCCAGGTGTGAATCCAGGAGTGG Rv: CCACTCCTGGATTCACACCTGGATTTGTCCAGTGCCATCT
K248STOP	Fw: TGCCTTCAAATCTCTTGACTGAGATGGCACTGGACAAATC Rv: GATTTGTCCAGTGCCATCTCAGTCAAGAGATTTGAAGGCA
M224STOP	Fw: TCAGATGAAAGTGGGAACTGAGATTTTGACAACTTCATCAGCTGCT Rv: CCAAGCAGCTGATGAAGTTGTCAAAATCTCAGTTCCCACTTTCATC

### Mammalian cell culture, immunoblotting, and casein zymography

HEK293T (American Type Culture Collection; #CRL-3216) and MDA-MB-231 (American Type Culture Collection; #HTB-26) cells were grown at 37 °C and 5% CO_2_ in complete Dulbecco's modified Eagle's medium (Gibco; catalog no.: 12100-061), 10% fetal bovine serum (Corning; catalog no.: 35-077-CV), 1% 200 mM l-glutamine (Corning; catalog no.: 25-005-CI), and 1% penicillin–streptomycin mix (Gibco; catalog no.: 15240-062) on tissue culture–treated 10-cm plates (Sarstedt; catalog no.: 83.3902). Plasmid transfections were done in 12-well plates (Sarstedt; catalog no.: 83.3921) using PolyJet transfection reagent (SignaGen; catalog no.: SL100688) in accordance with their protocol using 250 ng of plasmid DNA per well. Retroviruses were made by cotransfecting HEK293T cells cultured in 10-cm plates, using 8 mg of pMSCV_CAPNS1 (WT or mutant versions), and 8 mg of pCL-Ampho (Novus Biologicals; catalog no.: NBP2-29541) according to the PolyJet protocol. The cell cultures were routinely tested for mycoplasma contamination, similarly to (48), and prophylactically maintained in ciprofloxacin-containing media before and after freeze–thaw cycles.

CRISPR–Cas9 gene KOs were achieved by infecting MDA-MB-231 cells with lentivirus formed in HEK293T cells from psPAX2, pMD.2G, and lentiCRISPRv2 (49) plasmids. The gene-targeting guide RNA sequence and the respective primers cloned into BsmBI site of lentiCRISPRv2 are shown below.

**Table:** 

gRNA + **PAM sequence**	5′-GGCGGCTGCGCAGTACAACC**CGG**-3′
Fw primer	5′-CACCG GGCGGCTGCGCAGTACAACC-3′
Rv primer	3′-CCCGCCGACGCGTCATGTTGG CAAA-5′

Soluble cell lysates for immunoblotting were made in the kinase lysis buffer (20 mM Tris–HCl [pH 7.5], 150 mM NaCl, 1 mM EDTA, 1% v/v Nonidet P-40, and 0.5% w/v sodium deoxycholic acid), supplemented with 100 μM PMSF, 10 ng/ml leupeptin, 100 μM aprotinin, and 100 μM sodium orthovanadate. Antibodies used are Calpain 2 Large Subunit (M-type) Antibody (catalog no.: 2539S; Cell Signaling), α-Tubulin Antibody (catalog no.: 2144; Cell Signaling Technology), and anti–calpain small subunit antibody clone P-1 (MAB3083; MilliporeSigma). The specificity of antibodies against CAPN2 and CAPNS1 was verified on a panel of CAPN1, CAPN2, and CAPNS1 CRISPR–Cas9 KO and Rescue MDA-MB-231 cell lines (data not shown).

Hepes–imidazole casein zymogram was done in accordance with the primary publication (50), using kinase lysis buffer and 10% gels.

Soluble cell lysates for the luciferase reaction were made using 5x Passive Lysis Buffer (Promega; catalog no.: E1941), according to the product recommendations.

### Protein expression and purification

BL21 RIPL *E. coli* (Agilent; catalog no.: 230280), heat shock–transformed with pET16b/pHT29 plasmids expressing CAPNS1, CAPN1, or CAPN2 biosensor components, were grown in 2xYT media (7.5 g/l yeast extract, 20 g/l tryptone, 25 g/l LB Miller) until absorbance reached 0.6 at 600 nm, at which point protein expression was induced with 0.3 mM IPTG and continued for 16 h at 20 °C. Cells were sedimented, washed, and then lysed by sonication in 100 ml of standard buffer (30 mM Tris–HCl [pH 7.5], 150 mM NaCl, 3 mM 2-mercaptoethanol, and 5 mM CaCl_2_) per 1 l of liquid culture, supplemented with lysozyme and DNAse I. After that, the insoluble debris was removed by centrifugation at 20,000 RCF 4 °C for 30 min. Recombinant protein from the resulting soluble lysate was column-purified using 1 ml HisTrap HP columns (Cytiva; catalog no.: 17524701) in accordance with the manufacturer's recommendations, using 20 mM imidazole for washes and 250 mM imidazole for the elution. The eluted proteins were then dialyzed in the standard buffer (at a 1:400 ratio or greater) to remove imidazole, after which protein concentrations were determined relative to a bovine serum albumin standard, and the purified proteins were snap-frozen in multiple aliquots.

#### NanoBiT assay

Biosensor components were mixed and preincubated for 30 to 60 min in 45 μl of the standard buffer, unless otherwise specified, followed by addition of 5 μl of 1:50 diluted NanoGlo Live Substrate (Promega; catalog no.: N2011) and another 15-min incubation. After that, luminescence was measured using GloMax Navigator 96-well plate luminometer at 0.3 s integration setting.

#### Native state protein assays

LgBiT (10 μg) and SmBiT (100 μg) biosensor mix was run on a HiLoad 16/60 Superdex 200 size-exclusion chromatography column (Cytiva). UV absorbance was monitored at 280 nm and plotted against the retention volume using an Äkta start protein purification system (Cytiva).

LgBiT:SmBiT biosensor mixes (250 nM:2500 nM or 2500 nM:250 nM) were used for native protein polyacrylamide gel electrophoresis with Native PAGE 3 to 12% Bis–Tris gels (Invitrogen), which were Coomassie stained or immunoblotted with anti–calpain small subunit antibody clone P-1 (MAB3083; MilliporeSigma) or an anti-MBP antibody (NEB; #E8032L).

### Data analysis

Statistical analyses, nonlinear curve fitting, and data visualization were performed with GraphPad Prism 9 (GraphPad Software, Inc). Data are represented as mean ± standard deviation. Normality of datasets was verified by Shapiro–Wilk test (*p* < 0.05) where appropriate. Comparative statistics are conducted as one-way ANOVA tests with an uncorrected Fisher least significant difference follow-up test where necessary. Protein models are visualized with PyMOL Molecular Graphics System, version 2.5.2 (Schrödinger, LLC).

### Computational modeling of effect of mutations on calpain heterodimerization

We aimed to identify the most deleterious mutations for the interfacial residues of human calpain-2 (PDB ID: 1KFX) that compromise the binding affinity but not protein folding itself. We began by identifying the interfacial residues based on solvent accessible surface area (SASA). The change in SASA (ΔSASA) was calculated by comparing the SASA of the free state (SASA_free_) with the SASA of the bound state (SASA_bound_). Residues with a nonzero (ΔSASA) were classified as interfacial, indicating their involvement in the interaction interface. If ΔSASA ≠ 0, then the residue is interfacial where

ΔSASA = SASA_free_ – SASA_bound_

Alanine scanning mutagenesis was performed for all the interfacial residues. The binding free energy change upon mutation ΔΔG_binding_ was predicted using six prediction tools, namely: SAAMBE-3D (51), SAAMBE-SEQ (52), mCSM-PPI2 (53), BeAtMuSiC (54), MutaBind2 (55), and SSIPe (56). To ensure that the selected mutations did not adversely impact the overall protein stability, we computed the folding free energy change upon mutation ΔΔG_folding_ using six webservers: PoPMuSiC (57), INPS-MD (58), I-Mutant2 (59), mCSM (60), Site Directed Mutator (SDM) (61), and DUET (62). Six different methods or web servers used for prediction of binding free energy change and folding free energy change each are described below.

#### Binding free energy change prediction tools

a. SAAMBE-3D (51) (http://compbio.clemson.edu/saambe_webserver/index3D.php):

SAAMBE-3D is a structure-based machine learning tool, specifically trained using the XGBoost algorithm on a dataset of 3753 single-point mutations from the SKEMPI v2.0 database. It predicts binding free energy changes upon mutation and is highly accurate, with a Pearson correlation coefficient of 0.8.

b. SAAMBE-SEQ (52) (http://compbio.clemson.edu/saambe_webserver/indexSEQ.php):

SAAMBE-SEQ is a sequence-based tool that uses the Gradient Boosting Decision Tree algorithm to predict changes in binding free energy because of mutations. This tool is useful for large-scale studies and does not require structural data.

c. mCSM-PPI2 (53) (http://biosig.unimelb.edu.au/mcsm_ppi2):

mCSM-PPI2 is a structure-based tool that predicts how mutations affect PPIs using an optimized graph-based signature approach. It integrates atomic distance patterns with evolutionary and energetic information to predict the effects of mutations on binding affinity.

d. BeAtMuSiC (54) (http://babylone.ulb.ac.be/beatmusic):

BeAtMuSiC is a structure-based coarse-grained predictor tool of changes in binding free energy caused by point mutations. It uses statistical potentials derived from known protein structures to estimate how mutations affect the strength of interactions at the interface and the overall stability of the protein complex.

e. MutaBind2 (55) (https://lilab.jysw.suda.edu.cn/research/mutabind2/):

MutaBind2 is a structure-based tool developed using the SKEMPI v2.0 database to predict changes in binding affinity because of mutations. It utilizes just seven key features, with the most significant ones capturing the interaction of proteins with the solvent, the evolutionary conservation of the mutation site, and the thermodynamic stability of both the protein complex and its individual monomers.

f. SSIPe (56) (https://zhanggroup.org/SSIPe/):

SSIPe is a structure-based tool that predicts changes in binding affinity for PPIs because of mutations at the interface. It integrates structural and sequence alignments from PDB and STRING databases with the EvoEF energy function to assess the impact of mutations.

#### Folding free energy change prediction tools:

a. PoPMuSiC (63) (http://soft.dezyme.com):

PoPMuSiC is a computational tool designed to predict the stability changes in proteins caused by single-site mutations. It uses linear combinations of database-derived potentials, adjusting the coefficients based on the solvent accessibility of the mutated residues. The program computes all possible mutations in a given protein structure and ranks them as stabilizing, destabilizing, or neutral based on their predicted impact on folding free energy.

b. INPS-MD-3D (58) (http://inpsmd.biocomp.unibo.it):

INPS-3D is a structure-based tool within the INPS-MD server, designed to predict the stability changes in proteins caused by single-point mutations. It uses features derived from both the protein's 3D structure and sequence, incorporating descriptors like relative solvent accessibility and local energy difference between the WT and mutated structures.

c. I-Mutant2 (59) (http://folding.biofold.org/i-mutant/i-mutant2.0.html):

I-Mutant2.0 is a support vector machine–based tool that predicts protein stability changes upon single-point mutations, using either protein sequence or structure as input. The tool was trained on a dataset from the ProTherm database and can be used both as a classifier and regressor for predicting the sign and value of stability changes, respectively. The structure-based method was used in our case.

d. mCSM (60) (http://biosig.unimelb.edu.au/mcsm):

mCSM is a structure-based tool that employs graph-based signatures to model atomic distance patterns. It predicts how mutations affect protein stability by encoding these distance patterns, allowing for insights into folding free energy changes.

e. SDM (61) (http://structure.bioc.cam.ac.uk/sdm2, non-operational now):

SDM (Site Directed Mutator) is a structure-based tool that uses environment-specific substitution tables to predict the effects of mutations on protein stability. It compares the structural environments of the WT and mutant residues to estimate ΔΔG_folding_.

f. DUET (62) (http://biosig.unimelb.edu.au/duet):

DUET is a structure-based tool that combines predictions from two complementary methods, mCSM and SDM, to predict protein stability changes upon mutation. It provides more accurate ΔΔG_folding_ predictions by leveraging the strengths of both approaches.

## Data availability

All data described are contained within the article or available as supporting information. Plasmids, proteins, and cell lines will be made available upon request to the corresponding author.

## Supporting information

This article contains supporting information.

## CRediT authorship contribution statement

**Ivan Shapovalov:** Writing – original draft, Visualization, Methodology, Investigation, Conceptualization. **Prawin Rimal:** Writing – original draft, Software, Methodology, Investigation, Formal analysis, Data curation, Conceptualization. **Pitambar Poudel:** Writing – original draft, Software, Methodology, Investigation, Data curation, Conceptualization. **Victoria Lewtas:** Validation, Investigation, Formal analysis. **Mathias Bell:** Formal analysis, Investigation, Validation. **Shailesh Kumar Panday:** Validation, Formal analysis. **Brian J. Laight:** Validation, Investigation. **Danielle Harper:** Validation, Investigation. **Stacy Grieve:** Methodology, Investigation. **George S. Baillie:** Methodology, Investigation. **Kazem Nouri:** Methodology, Investigation, Conceptualization. **Peter L. Davies:** Writing – review & editing. **Emil Alexov:** Writing – review & editing, Supervision, Resources, Project administration, Funding acquisition, Conceptualization. **Peter A. Greer:** Writing – review & editing, Supervision, Resources, Project administration, Funding acquisition, Conceptualization.

## Conflict of interest

The authors declare that they have no conflicts of interest with the contents of this article.
