# Supplementary material for: Quantification and structure–function analysis of calpain-1 and calpain-2 protease subunit interactions
Source: J Biol Chem. 2025 May 16;301(6):110243. doi: 10.1016/j.jbc.2025.110243 (PMC12205653; doi:10.1016/j.jbc.2025.110243)
Supplement: Supporting information [file mmc1.docx]

**Title**

Quantification and structure-function analysis of calpain-1 and calpain-2 protease subunit interactions

**Authors**

Ivan Shapovalov^1,2,&^, Prawin Rimal^3,&^, Pitambar Poudel^3^, Victoria Lewtas^1,2^, Mathias Bell^4^, Shailesh Kumar Panday^3^, Brian J. Laight^1,2^, Danielle Harper^1,2^, Stacey Grieve^2^, George S. Baillie^5^, Kazem Nouri^6^, Peter L. Davies^4^, Emil Alexov^3^, Peter A. Greer^1,2,*^

^1^Dept Pathology and Molecular Medicine, School of Medicine, Queen’s University, Kingston, ON, Canada

^2^ Division of Cancer Biology and Genetics, Sinclair Cancer Research Institute, Queen's University, Kingston, Ontario, Canada

^3^ Department of Physics, College of Science, Clemson University, Clemson, South Carolina, USA

^4^ Department of Biomedical and Molecular Sciences, Queen's University, Kingston, Ontario, Canada

^5^ School of Cardiovascular and Metabolic Health, University of Glasgow, Glasgow, Scotland, UK

^6^ Department of Pathology and Laboratory Medicine, University of British Columbia, Vancouver, British Columbia, Canada

*Corresponding author ^&^Co-first authors

**List of the material included**

| **Figure S1.** **Bacterial expression of CAPNS1, CAPN1 and CAPN2 biosensor components** | **Page S-2** |
| --- | --- |
| **Figure S2.** **Peptide array analysis of CAPNS1 binding to CAPN1 and CAPN2** | **Page S-3** |
| **Figure S3.** **Z-score analysis of the CAPNS1-CAPN1 biosensor** | **Page S-4** |
| **Figure S4. Native state protein-protein interactions** | **Page S-5** |
| **Table S1. The binding free energy values obtained from 6 webservers for stated mutations at the interfacial residues of human calpain-2** | **Page S-6** |
| **Table S2. The folding free energy values obtained from 6 webservers for stated mutations at the interfacial residues of human calpain-2** | **Page S-7** |
| **Table S3. His-MBP-SmBiT-CAPN1 DNA coding sequence, amino acid sequence, and annotations.** | **Page S-8** |
| **Table S4. His-MBP-SmBiT-CAPN2 DNA coding sequence, amino acid sequence, and annotations.** | **Page S-11** |
| **Table S5. His-LgBiT-CAPNS1 DNA coding sequence, amino acid sequence, and annotations.** | **Page S-14** |
| **Table S6. His-MBP-SmBiT-CAPNS1 DNA coding sequence, amino acid sequence, and annotations.** | **Page S-16** |
| **Table S7. Clustal multiple sequence alignment of CAPNS1, CAPN1, CAPN2 PEF domains.** | **Page S-19** |


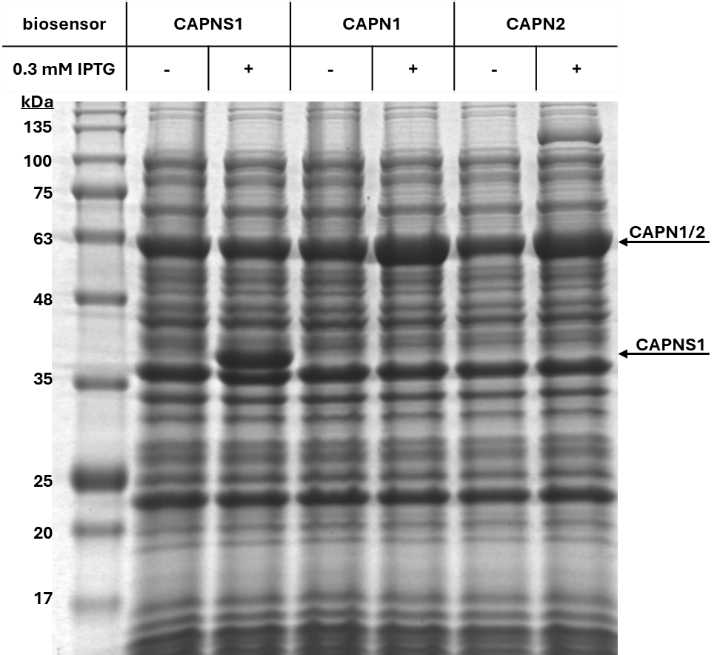


**Figure S1.** **Bacterial expression of CAPNS1, CAPN1 and CAPN2 biosensor components.** Coomassie stained SDS-PAGE of the soluble fractions of BL21 RIPL *E. coli* lysates before and after IPTG induction of protein expression shows excellent expression of the biosensor components.


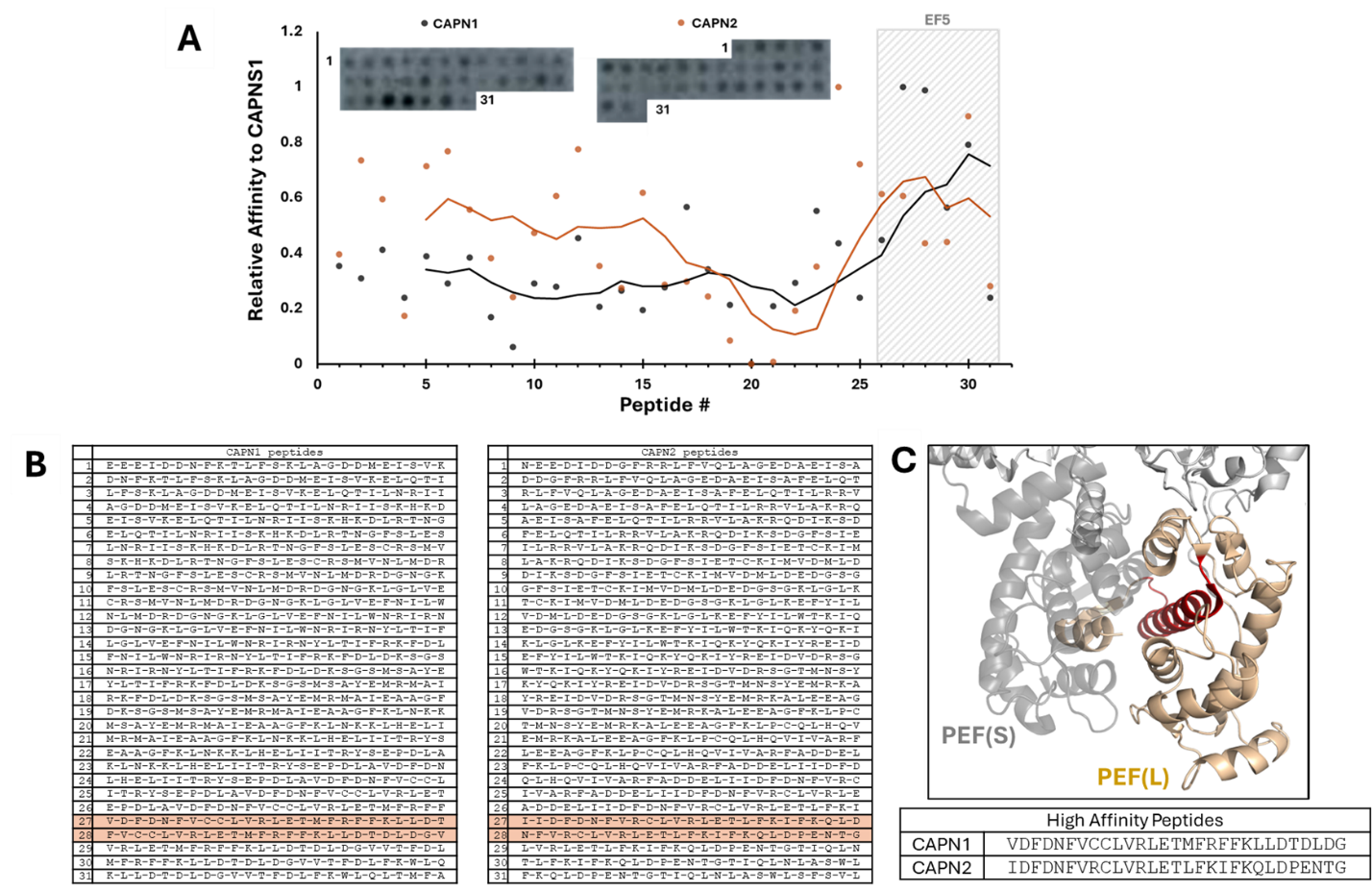


**Figure S2.** **Peptide array analysis of CAPNS1 binding to CAPN1 and CAPN2.** Peptide arrays consisting of overlapping 25mers from the PEF domains of human CAPN1 and CAPN2, were incubated with lysates from transfected HEK293 cells expressing recombinant Myc-tagged mouse CAPNS1 and subsequently probed with an HRP-linked anti-Myc epitope antibody, then detected by ECL. (**A**) Peptide array results for CAPN1 (black) and CAPN2 (brown) with densitometry quantitation. The blots were quantified using ImageJ. The resulting five-point moving average graphs indicate strongest binding to CAPN1 and CAPN2 peptides including the EF5 motif (shaded). (**B**) List of overlapping 25mers, numbered 1 through 31, covering residues 538-714, and 524-700 of human CAPN1 and CAPN2. (**C**) Structural representation of calpain-2 with the PEF(S) domain shown in light grey, the PEF(L) domain of CAPN2 in light brown, and the high affinity binding peptide region corresponding to portions of each of the EF4 and EF5 motifs shown in dark brown. The sequences of these highest affinity CAPN1 and CAPN2 binding peptides, (corresponding to this EF4/EF5 junction region), are shown below.


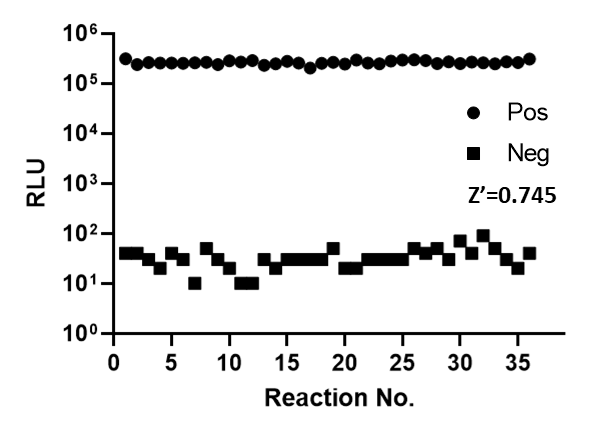


**Figure S3.** **Z-score analysis of the CAPNS1-CAPN1 biosensor.** Equal concentrations (25 nM each of SmBiT-CAPN1 and LgBiT-CAPNS1) of the purified CAPNS1-CAPN1 biosensor components were used in the optimal NanoBiT assay conditions described in the Material and Methods. The luminescence signals for negative controls (LgBiT-CAPNS1 alone – lower values between 10^1^ and 10^2^ relative light units) and the complete biosensor (upper values between 10^5^ and 10^6^ RLU) were highly reproducible with a large dynamic range and a Z-factor of 0.745. This was similar to the Z-score achieved with the CAPNS1-CAPN2 biosensor (Figure 4F).


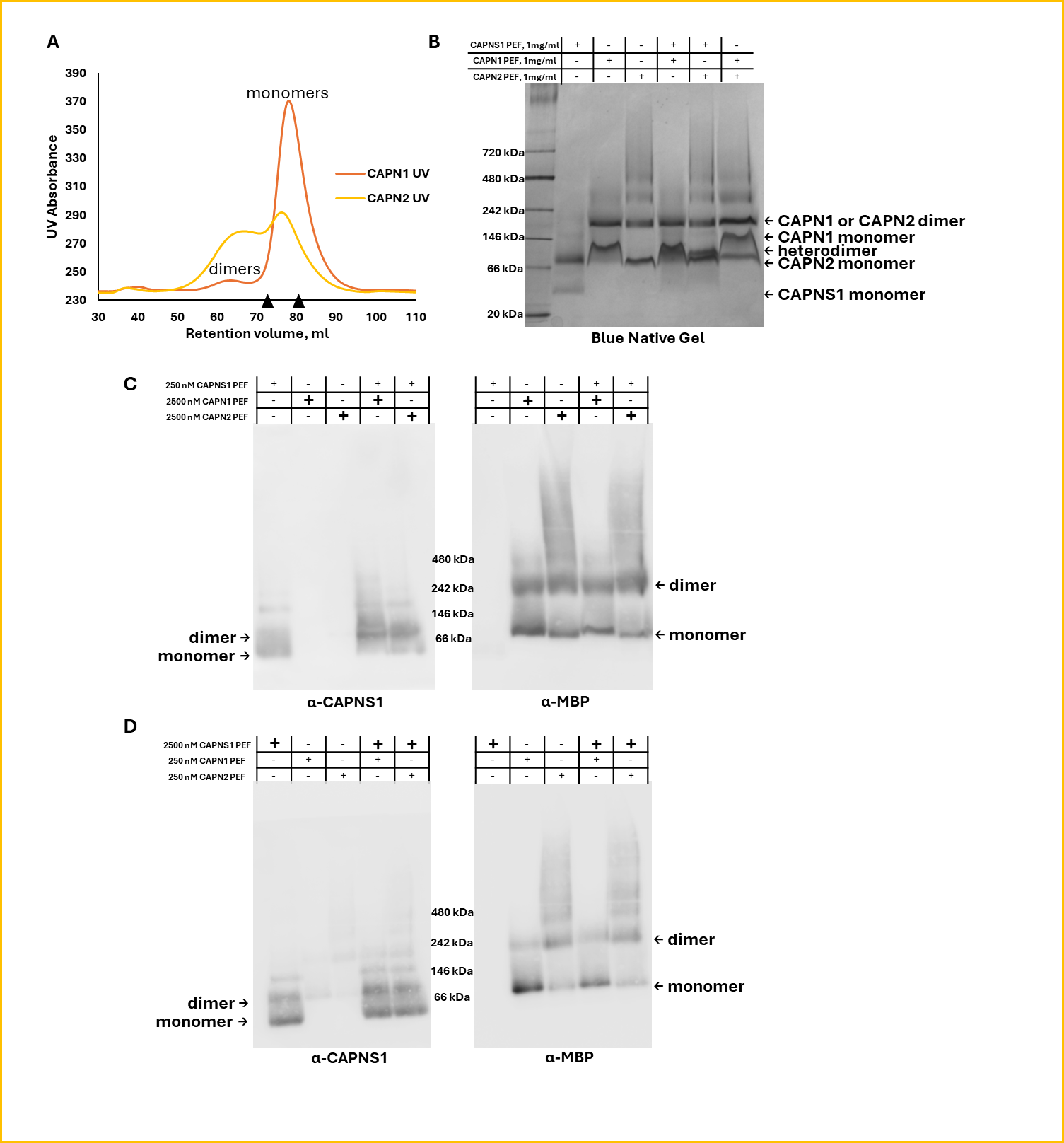


**Figure S4.** **Native state protein-protein interactions.** (**A**) S200 size exclusion chromatography of premixed calpain-1 and calpain-2 biosensor components. Black arrows indicate elution volumes of 75kDa (left) and 44kDa (right) standards. (**B**) Blue native PAGE of preincubated biosensor component mixtures, as indicated. Depletion of the CAPNS1 monomer, presumably drawn into heterodimers by CAPN1 and CAPN2 PEFs, and the emergence of the heterodimer band are apparent. (**C, D**) Immunoblot analysis of blue native PAGE of (**C**) a 10:1 concentration ratio of CAPN1/2:CAPNS1 mixtures shows depletion of CAPNS1 monomers (detected with α-CAPNS1, left panel) relative to presumptive heterodimers, while CAPN1/CAPN2 monomers (detected with α-MBP) remain relatively unchanged. In contrast, when CAPNS1 is in 10:1 excess relative to CAPN1/CAPN2 (**D**), CAPNS1 monomers remain unchanged with addition of CAPN1/2, while CAPN1 and CAPN2 monomer levels are depleted. Immunoblotting in panels (C) and (D) used anti-CAPNS1 (MAB3083, Millipore Sigma) and anti-MBP antibody (NEB #E8032L).

**Table S1. The binding free energy values obtained from 6 webservers for stated mutations at the interfacial residues of human calpain-2.**

| **Subunit** | **Position** | **Residue** | **SAAMBE-3D** | **SAAMBE-SEQ** | **mCSM-PPI2** | **BeAtMuSiC** | **MutaBind2** | **SSIPe** | **Average** |
| --- | --- | --- | --- | --- | --- | --- | --- | --- | --- |
|  |  |  | **ΔΔG_binding_, kcal/mol** | | | | | | |
| CAPN2 | 417 | R | 0.60 | 1.40 | 0.44 | 0.88 | 1.83 | 1.78 | 1.15 |
| CAPNS1 | 154 | D | 0.97 | 0.59 | 0.93 | 1.10 | 0.76 | 1.45 | 0.97 |
| CAPNS1 | 111 | D | 0.80 | 0.99 | 1.06 | 2.50 | 0.18 | 0.59 | 1.02 |
| CAPNS1 | 112 | D | 1.11 | 0.73 | 0.63 | 1.26 | 0.65 | 1.36 | 0.96 |
| CAPNS1 | 263 | Q | 0.75 | 0.41 | 0.85 | 1.90 | 0.81 | 1.29 | 1.00 |
| CAPNS1 | 118 | T | 0.36 | 0.75 | 0.45 | 1.42 | 1.50 | 1.26 | 0.96 |
| CAPN2 | 690 | D | 1.11 | 0.85 | 0.78 | -0.07 | 0.90 | 0.53 | 0.68 |
| CAPN2 | 653 | D | 0.88 | 0.46 | 0.63 | 1.78 | 0.66 | 0.64 | 0.84 |
| CAPN2 | 7 | K | 0.90 | 0.43 | 0.63 | 1.04 | 0.95 | 0.41 | 0.73 |
| CAPNS1 | 119 | E | 0.47 | 0.75 | 0.63 | 1.42 | 0.44 | 1.33 | 0.84 |
| CAPN2 | 367 | R | 0.57 | 1.10 | 0.55 | 0.55 | 0.67 | 1.08 | 0.75 |
| CAPNS1 | 265 | T | 0.55 | 0.58 | 0.37 | 0.86 | 0.88 | 1.57 | 0.80 |
| CAPN2 | 418 | R | 0.87 | 0.39 | 0.52 | 0.85 | 0.72 | 1.46 | 0.80 |
| CAPN2 | 420 | R | 0.50 | 0.98 | 0.36 | 0.44 | 0.72 | 1.46 | 0.74 |
| CAPN2 | 582 | D | 0.94 | 0.49 | 0.62 | 1.64 | 0.00 | 0.84 | 0.76 |
| CAPNS1 | 155 | T | 0.45 | 0.98 | 0.65 | 0.35 | 0.31 | 1.66 | 0.73 |
| CAPN2 | 575 | E | 0.75 | 0.45 | 0.59 | 1.49 | 0.37 | 1.07 | 0.79 |
| CAPNS1 | 163 | E | 0.51 | 0.40 | 0.88 | 0.69 | 0.53 | 1.56 | 0.76 |
| CAPN2 | 661 | N | 0.14 | 0.59 | 0.99 | 0.73 | 0.59 | 1.08 | 0.69 |
| CAPN2 | 494 | D | 0.64 | 0.42 | 0.78 | 0.29 | 1.03 | 0.42 | 0.60 |

**Table S2. The folding free energy values obtained from 6 webservers for stated mutations at the interfacial residues of human calpain-2.**

| **Subunit** | **Position** | **Residue** | **PoPMuSiC** | **INPS-3D** | **I-Mutant 2** | **mCSM** | **SDM** | **DUET** | **Average** | **Absolute**  **Average** |
| --- | --- | --- | --- | --- | --- | --- | --- | --- | --- | --- |
|  |  |  | **ΔΔG_binding_, kcal/mol** | | | | | | | |
| CAPN2 | 417 | R | -0.66 | -0.47 | -0.87 | -0.07 | 0.11 | 0.04 | -0.32 | 0.32 |
| CAPNS1 | 154 | D | -0.58 | -0.31 | -0.35 | -0.14 | -0.27 | 0.03 | -0.27 | 0.27 |
| CAPNS1 | 111 | D | -0.20 | -0.44 | 0.41 | -0.31 | 0.52 | 0.10 | 0.02 | 0.02 |
| CAPNS1 | 112 | D | -1.50 | -0.41 | 0.01 | -0.26 | 1.80 | 0.42 | 0.01 | 0.01 |
| CAPNS1 | 263 | Q | -1.40 | 0.20 | -0.95 | -1.21 | 1.21 | -0.59 | -0.46 | 0.46 |
| CAPNS1 | 118 | T | -0.91 | 0.02 | -0.86 | -0.78 | 1.13 | -0.17 | -0.26 | 0.26 |
| CAPN2 | 690 | D | -0.35 | 0.16 | -0.94 | 0.15 | 0.03 | 0.48 | -0.08 | 0.08 |
| CAPN2 | 653 | D | -1.25 | -0.15 | -1.55 | -0.20 | 0.19 | 0.08 | -0.48 | 0.48 |
| CAPN2 | 7 | K | -1.17 | 0.66 | -0.91 | -0.32 | 1.26 | 0.28 | -0.03 | 0.03 |
| CAPNS1 | 119 | E | -0.59 | -0.32 | -0.07 | -0.80 | 0.57 | -0.38 | -0.27 | 0.27 |
| CAPN2 | 367 | R | -0.83 | -0.68 | -0.75 | -0.87 | 0.77 | -0.51 | -0.48 | 0.48 |
| CAPNS1 | 265 | T | -0.87 | -0.88 | -0.67 | -1.14 | 1.93 | -0.32 | -0.32 | 0.32 |
| CAPN2 | 418 | R | -0.84 | -0.36 | -0.93 | -0.73 | 0.59 | -0.42 | -0.45 | 0.45 |
| CAPN2 | 420 | R | -0.59 | -0.54 | -0.95 | 0.05 | 0.20 | 0.19 | -0.27 | 0.27 |
| CAPN2 | 582 | D | -1.06 | 0.24 | -1.60 | -0.58 | 1.92 | 0.18 | -0.15 | 0.15 |
| CAPNS1 | 155 | T | -0.95 | 0.00 | -0.26 | -0.90 | 0.64 | -0.51 | -0.33 | 0.33 |
| CAPN2 | 575 | E | -1.59 | -0.48 | -0.20 | -0.75 | 0.73 | -0.28 | -0.43 | 0.43 |
| CAPNS1 | 163 | E | -0.74 | -0.41 | -0.30 | -0.76 | 0.90 | -0.25 | -0.26 | 0.26 |
| CAPN2 | 661 | N | -1.10 | -0.27 | -2.09 | -1.02 | 1.83 | -0.19 | -0.47 | 0.47 |
| CAPN2 | 494 | D | -0.80 | -0.52 | -1.34 | -0.29 | 0.67 | 0.09 | -0.36 | 0.36 |

**Table S3. His-MBP-SmBiT-CAPN1 DNA coding sequence, amino acid sequence, and annotations.**

**Sequence Annotation**

1 ATGGGCCATCATCATCATCATCATGGCATGAAAATCGAAGAAGGT His-tag MBP start

M G H H H H H H G M K I E E G

46 AAACTGGTAATCTGGATTAACGGCGATAAAGGCTATAACGGTCTC

K L V I W I N G D K G Y N G L

91 GCTGAAGTCGGTAAGAAATTCGAGAAAGATACCGGAATTAAAGTC

A E V G K K F E K D T G I K V

136 ACCGTTGAGCATCCGGATAAACTGGAAGAGAAATTCCCACAGGTT

T V E H P D K L E E K F P Q V

181 GCGGCAACTGGCGATGGCCCTGACATTATCTTCTGGGCACACGAC

A A T G D G P D I I F W A H D

226 CGCTTTGGTGGCTACGCTCAATCTGGCCTGTTGGCTGAAATCACC

R F G G Y A Q S G L L A E I T

271 CCGGACAAAGCGTTCCAGGACAAGCTGTATCCGTTTACCTGGGAT

P D K A F Q D K L Y P F T W D

316 GCCGTACGTTACAACGGCAAGCTGATTGCTTACCCGATCGCTGTT

A V R Y N G K L I A Y P I A V

361 GAAGCGTTATCGCTGATTTATAACAAAGATCTGCTGCCGAACCCG

E A L S L I Y N K D L L P N P

406 CCAAAAACCTGGGAAGAGATCCCGGCGCTGGATAAAGAACTGAAA

P K T W E E I P A L D K E L K

451 GCGAAAGGTAAGAGCGCGCTGATGTTCAACCTGCAAGAACCGTAC

A K G K S A L M F N L Q E P Y

496 TTCACCTGGCCGCTGATTGCTGCTGACGGGGGTTATGCGTTCAAG

F T W P L I A A D G G Y A F K

541 TATGAAAACGGCAAGTACGACATTAAAGACGTGGGCGTGGATAAC

Y E N G K Y D I K D V G V D N

586 GCTGGCGCGAAAGCGGGTCTGACCTTCCTGGTTGACCTGATTAAA

A G A K A G L T F L V D L I K

631 AACAAACACATGAATGCAGACACCGATTACTCCATCGCAGAAGCT

N K H M N A D T D Y S I A E A

676 GCCTTTAATAAAGGCGAAACAGCGATGACCATCAACGGCCCGTGG

A F N K G E T A M T I N G P W

721 GCATGGTCCAACATCGACACCAGCAAAGTGAATTATGGTGTAACG

A W S N I D T S K V N Y G V T

766 GTACTGCCGACCTTCAAGGGTCAACCATCCAAACCGTTCGTTGGC

V L P T F K G Q P S K P F V G

811 GTGCTGAGCGCAGGTATTAACGCCGCCAGTCCGAACAGAGAGCTG

V L S A G I N A A S P N R E L

856 GCAAAAGAGTTCCTCGAAAACTATCTGCTGACTGATGAAGGTCTG

A K E F L E N Y L L T D E G L

901 GAAGCGGTTAATAAAGACAAACCGCTGGGTGCCGTAGCGCTGAAG

E A V N K D K P L G A V A L K

946 TCTTACGAGGAAGAGTTGGCGAAAGATCCACGTATTGCCGCCACT

S Y E E E L A K D P R I A A T

991 ATGGAAAACGCCCAGAAAGGTGAAATCATGCCGAACATCTCGCAG

M E N A Q K G E I M P N I S Q

1036 ATGTCCGCTTTCTGGTATGCCGTGCGTACTGCGGTGATCAACGCC

M S A F W Y A V R T A V I N A

1081 GCCAGCGGTCGTCAGACTGTCGATGCAGCCCTGAAAGACGCGCAG

A S G R Q T V D A A L K D A Q

1126 ACTAATTCGCCGTCCGGCGGCGAAAACCTGTATTTTCAGTCTGGA MBP end

T N S P S G G E N L Y F Q S G

1171 GGATCCCATATGATGGTGACCGGCTACCGGCTGTTCGAGGAGATT SmBiT

G S H M M V T G Y R L F E E I

1216 CTCGGGAGTTCCGGTGGTGGCGGGAGCGGAGGTGGAGGCTCGAGC GS-linker

L G S S G G G G S G G G G S S

1261 CTCTCAGAAGAGGAGATTGACGAGAACTTCAAGGCCCTCTTCAGG CAPN1 start

L S E E E I D E N F K A L F R

1306 CAGCTGGCAGGGGAGGACATGGAGATCAGCGTGAAGGAGTTGCGG

Q L A G E D M E I S V K E L R

1351 ACAATCCTCAATAGGATCATCAGCAAACACAAAGACCTGCGGACC

T I L N R I I S K H K D L R T

1396 AAGGGCTTCAGCCTAGAGTCGTGCCGCAGCATGGTGAACCTCATG

K G F S L E S C R S M V N L M

1441 GATCGTGATGGCAATGGGAAGCTGGGCCTGGTGGAGTTCAACATC

D R D G N G K L G L V E F N I

1486 CTGTGGAACCGCATCCGGAATTACCTGTCCATCTTCCGGAAGTTT

L W N R I R N Y L S I F R K F

1531 GACCTGGACAAGTCGGGCAGCATGAGTGCCTACGAGATGCGGATG

D L D K S G S M S A Y E M R M

1576 GCCATTGAGTCGGCAGGCTTCAAGCTCAACAAGAAGCTGTACGAG

A I E S A G F K L N K K L Y E

1621 CTCATCATCACCCGCTACTCGGAGCCCGACCTGGCGGTCGACTTT

L I I T R Y S E P D L A V D F

1666 GACAATTTCGTTTGCTGCCTGGTGCGGCTAGAGACCATGTTCCGA

D N F V C C L V R L E T M F R

1711 TTTTTCAAAACTCTGGACACAGATCTGGATGGAGTTGTGACCTTT

F F K T L D T D L D G V V T F

1756 GACTTGTTTAAGTGGTTGCAGCTGACCATGTTTGCATAA stop codon, CAPN1 end

D L F K W L Q L T M F A *

**Table S4. His-MBP-SmBiT-CAPN2 DNA coding sequence, amino acid sequence, and annotations.**

**Sequence Annotation**

1 ATGGGCCATCATCATCATCATCATGGCATGAAAATCGAAGAAGGT His-tag MBP start

M G H H H H H H G M K I E E G

46 AAACTGGTAATCTGGATTAACGGCGATAAAGGCTATAACGGTCTC

K L V I W I N G D K G Y N G L

91 GCTGAAGTCGGTAAGAAATTCGAGAAAGATACCGGAATTAAAGTC

A E V G K K F E K D T G I K V

136 ACCGTTGAGCATCCGGATAAACTGGAAGAGAAATTCCCACAGGTT

T V E H P D K L E E K F P Q V

181 GCGGCAACTGGCGATGGCCCTGACATTATCTTCTGGGCACACGAC

A A T G D G P D I I F W A H D

226 CGCTTTGGTGGCTACGCTCAATCTGGCCTGTTGGCTGAAATCACC

R F G G Y A Q S G L L A E I T

271 CCGGACAAAGCGTTCCAGGACAAGCTGTATCCGTTTACCTGGGAT

P D K A F Q D K L Y P F T W D

316 GCCGTACGTTACAACGGCAAGCTGATTGCTTACCCGATCGCTGTT

A V R Y N G K L I A Y P I A V

361 GAAGCGTTATCGCTGATTTATAACAAAGATCTGCTGCCGAACCCG

E A L S L I Y N K D L L P N P

406 CCAAAAACCTGGGAAGAGATCCCGGCGCTGGATAAAGAACTGAAA

P K T W E E I P A L D K E L K

451 GCGAAAGGTAAGAGCGCGCTGATGTTCAACCTGCAAGAACCGTAC

A K G K S A L M F N L Q E P Y

496 TTCACCTGGCCGCTGATTGCTGCTGACGGGGGTTATGCGTTCAAG

F T W P L I A A D G G Y A F K

541 TATGAAAACGGCAAGTACGACATTAAAGACGTGGGCGTGGATAAC

Y E N G K Y D I K D V G V D N

586 GCTGGCGCGAAAGCGGGTCTGACCTTCCTGGTTGACCTGATTAAA

A G A K A G L T F L V D L I K

631 AACAAACACATGAATGCAGACACCGATTACTCCATCGCAGAAGCT

N K H M N A D T D Y S I A E A

676 GCCTTTAATAAAGGCGAAACAGCGATGACCATCAACGGCCCGTGG

A F N K G E T A M T I N G P W

721 GCATGGTCCAACATCGACACCAGCAAAGTGAATTATGGTGTAACG

A W S N I D T S K V N Y G V T

766 GTACTGCCGACCTTCAAGGGTCAACCATCCAAACCGTTCGTTGGC

V L P T F K G Q P S K P F V G

811 GTGCTGAGCGCAGGTATTAACGCCGCCAGTCCGAACAGAGAGCTG

V L S A G I N A A S P N R E L

856 GCAAAAGAGTTCCTCGAAAACTATCTGCTGACTGATGAAGGTCTG

A K E F L E N Y L L T D E G L

901 GAAGCGGTTAATAAAGACAAACCGCTGGGTGCCGTAGCGCTGAAG

E A V N K D K P L G A V A L K

946 TCTTACGAGGAAGAGTTGGCGAAAGATCCACGTATTGCCGCCACT

S Y E E E L A K D P R I A A T

991 ATGGAAAACGCCCAGAAAGGTGAAATCATGCCGAACATCTCGCAG

M E N A Q K G E I M P N I S Q

1036 ATGTCCGCTTTCTGGTATGCCGTGCGTACTGCGGTGATCAACGCC

M S A F W Y A V R T A V I N A

1081 GCCAGCGGTCGTCAGACTGTCGATGCAGCCCTGAAAGACGCGCAG

A S G R Q T V D A A L K D A Q

1126 ACTAATTCGCCGTCCGGCGGCGAAAACCTGTATTTTCAGTCTGGA MBP end

T N S P S G G E N L Y F Q S G

1171 GGATCCCATATGGTCGACATGGTGACCGGCTACCGGCTGTTCGAG SmBiT

G S H M V D M V T G Y R L F E

1216 GAGATTCTCGGGAGTTCCGGTGGTGGCGGGAGCGGAGGTGGAGGC GS-linker

E I L G S S G G G G S G G G G

1261 TCGAGCGACATCAGCGAGGATGACATTGATGATGGATTCAGGAGA CAPN2 start

S S D I S E D D I D D G F R R

1306 CTGTTTGCCCAGTTGGCAGGAGAGGATGCGGAGATCTCTGCCTTT

L F A Q L A G E D A E I S A F

1351 GAGCTGCAGACCATCCTGAGAAGGGTTCTAGCAAAGCGCCAAGAT

E L Q T I L R R V L A K R Q D

1396 ATCAAGTCAGATGGCTTCAGCATCGAGACATGCAAAATTATGGTT

I K S D G F S I E T C K I M V

1441 GACATGCTAGATTCGGACGGGAGTGGCAAGCTGGGGCTGAAGGAG

D M L D S D G S G K L G L K E

1486 TTCTACATTCTCTGGACGAAGATTCAAAAATACCAAAAAATTTAC

F Y I L W T K I Q K Y Q K I Y

1531 CGAGAAATCGACGTTGACAGGTCTGGTACCATGAATTCCTATGAA

R E I D V D R S G T M N S Y E

1576 ATGCGGAAGGCATTAGAAGAAGCAGGTTTCAAGATGCCCTGTCAA

M R K A L E E A G F K M P C Q

1621 CTCCACCAAGTCATCGTTGCTCGGTTTGCAGATGACCAGCTCATC

L H Q V I V A R F A D D Q L I

1666 ATCGATTTTGATAATTTTGTTCGGTGTTTGGTTCGGCTGGAAACG

I D F D N F V R C L V R L E T

1711 CTATTCAAGATATTTAAGCAGCTGGATCCCGAGAATACTGGAACA

L F K I F K Q L D P E N T G T

1756 ATAGAGCTCGACCTTATCTCTTGGCTCTGTTTCTCAGTACTTTAA stop codon, CAPN2 end

I E L D L I S W L C F S V L *

**Table S5. His-LgBiT-CAPNS1 DNA coding sequence, amino acid sequence, and annotations.**

**Sequence Annotation**

1 ATGGGCCATCATCATCATCATCATCATCATCATCACAGCAGCGGC His-tag LgBiT start

M G H H H H H H H H H H S S G

46 CATATCGAAGGTCGTCATATGGTCTTCACACTCGAAGATTTCGTT

H I E G R H M V F T L E D F V

91 GGGGACTGGGAACAGACAGCCGCCTACAACCTGGACCAAGTCCTT

G D W E Q T A A Y N L D Q V L

136 GAACAGGGAGGTGTGTCCAGTTTGCTGCAGAATCTCGCCGTGTCC

E Q G G V S S L L Q N L A V S

181 GTAACTCCGATCCAAAGGATTGTCCGGAGCGGTGAAAATGCCCTG

V T P I Q R I V R S G E N A L

226 AAGATCGACATCCATGTCATCATCCCGTATGAAGGTCTGAGCGCC

K I D I H V I I P Y E G L S A

271 GACCAAATGGCCCAGATCGAAGAGGTGTTTAAGGTGGTGTACCCT

D Q M A Q I E E V F K V V Y P

316 GTGGATGATCATCACTTTAAGGTGATCCTGCCCTATGGCACACTG

V D D H H F K V I L P Y G T L

361 GTAATCGACGGGGTTACGCCGAACATGCTGAACTATTTCGGACGG

V I D G V T P N M L N Y F G R

406 CCGTATGAAGGCATCGCCGTGTTCGACGGCAAAAAGATCACTGTA

P Y E G I A V F D G K K I T V

451 ACAGGGACCCTGTGGAACGGCAACAAAATTATCGACGAGCGCCTG

T G T L W N G N K I I D E R L

496 ATCACCCCCGACGGCTCCATGCTGTTCCGAGTAACCATCAACAGT LgBiT end GS-linker

I T P D G S M L F R V T I N S

541 GGGAGTTCCGGTGGTGGCGGGAGCGGAGGTGGAGGCTCGAGCCGC CAPNS1 start

G S S G G G G S G G G G S S R

586 ATCCTAGGCGGAGTCATCAGCGCCATCAGCGAGGCGGCTGCGCAG

I L G G V I S A I S E A A A Q

631 TACAACCCGGAGCCCCCGCCCCCACGCACACATTACTCCAACATT

Y N P E P P P P R T H Y S N I

676 GAGGCCAACGAGAGTGAGGAGGTCCGGCAGTTCCGGAGACTCTTT

E A N E S E E V R Q F R R L F

721 GCCCAGCTGGCTGGAGATGACATGGAGGTCAGCGCCACAGAACTC

A Q L A G D D M E V S A T E L

766 ATGAACATTCTCAATAAGGTTGTGACACGACACCCTGATCTGAAG

M N I L N K V V T R H P D L K

811 ACTGATGGTTTTGGCATTGACACATGTCGCAGCATGGTGGCCGTG

T D G F G I D T C R S M V A V

856 ATGGATAGCGACACCACAGGCAAGCTGGGCTTTGAGGAATTCAAG

M D S D T T G K L G F E E F K

901 TACTTGTGGAACAACATCAAAAGGTGGCAGGCCATATACAAACAG

Y L W N N I K R W Q A I Y K Q

946 TTCGACACTGACCGATCAGGGACCATTTGCAGTAGTGAACTCCCA

F D T D R S G T I C S S E L P

991 GGTGCCTTTGAGGCAGCAGGGTTCCACCTGAATGAGCATCTCTAT

G A F E A A G F H L N E H L Y

1036 AACATGATCATCCGACGCTACTCAGATGAAAGTGGGAACATGGAT

N M I I R R Y S D E S G N M D

1081 TTTGACAACTTCATCAGCTGCTTGGTCAGGCTGGACGCCATGTTC

F D N F I S C L V R L D A M F

1126 CGTGCCTTCAAATCTCTTGACAAAGATGGCACTGGACAAATCCAG

R A F K S L D K D G T G Q I Q

1171 GTGAACATCCAGGAGTGGCTGCAGCTGACTATGTATTCCTAA CAPNS1 end

V N I Q E W L Q L T M Y S *

**Table S6. His-MBP-SmBiT-CAPNS1 DNA coding sequence, amino acid sequence, and annotations.**

**Sequence Annotation**

1 ATGGGCCATCATCATCATCATCATGGCATGAAAATCGAAGAAGGT His-tag MBP start

M G H H H H H H G M K I E E G

46 AAACTGGTAATCTGGATTAACGGCGATAAAGGCTATAACGGTCTC

K L V I W I N G D K G Y N G L

91 GCTGAAGTCGGTAAGAAATTCGAGAAAGATACCGGAATTAAAGTC

A E V G K K F E K D T G I K V

136 ACCGTTGAGCATCCGGATAAACTGGAAGAGAAATTCCCACAGGTT

T V E H P D K L E E K F P Q V

181 GCGGCAACTGGCGATGGCCCTGACATTATCTTCTGGGCACACGAC

A A T G D G P D I I F W A H D

226 CGCTTTGGTGGCTACGCTCAATCTGGCCTGTTGGCTGAAATCACC

R F G G Y A Q S G L L A E I T

271 CCGGACAAAGCGTTCCAGGACAAGCTGTATCCGTTTACCTGGGAT

P D K A F Q D K L Y P F T W D

316 GCCGTACGTTACAACGGCAAGCTGATTGCTTACCCGATCGCTGTT

A V R Y N G K L I A Y P I A V

361 GAAGCGTTATCGCTGATTTATAACAAAGATCTGCTGCCGAACCCG

E A L S L I Y N K D L L P N P

406 CCAAAAACCTGGGAAGAGATCCCGGCGCTGGATAAAGAACTGAAA

P K T W E E I P A L D K E L K

451 GCGAAAGGTAAGAGCGCGCTGATGTTCAACCTGCAAGAACCGTAC

A K G K S A L M F N L Q E P Y

496 TTCACCTGGCCGCTGATTGCTGCTGACGGGGGTTATGCGTTCAAG

F T W P L I A A D G G Y A F K

541 TATGAAAACGGCAAGTACGACATTAAAGACGTGGGCGTGGATAAC

Y E N G K Y D I K D V G V D N

586 GCTGGCGCGAAAGCGGGTCTGACCTTCCTGGTTGACCTGATTAAA

A G A K A G L T F L V D L I K

631 AACAAACACATGAATGCAGACACCGATTACTCCATCGCAGAAGCT

N K H M N A D T D Y S I A E A

676 GCCTTTAATAAAGGCGAAACAGCGATGACCATCAACGGCCCGTGG

A F N K G E T A M T I N G P W

721 GCATGGTCCAACATCGACACCAGCAAAGTGAATTATGGTGTAACG

A W S N I D T S K V N Y G V T

766 GTACTGCCGACCTTCAAGGGTCAACCATCCAAACCGTTCGTTGGC

V L P T F K G Q P S K P F V G

811 GTGCTGAGCGCAGGTATTAACGCCGCCAGTCCGAACAGAGAGCTG

V L S A G I N A A S P N R E L

856 GCAAAAGAGTTCCTCGAAAACTATCTGCTGACTGATGAAGGTCTG

A K E F L E N Y L L T D E G L

901 GAAGCGGTTAATAAAGACAAACCGCTGGGTGCCGTAGCGCTGAAG

E A V N K D K P L G A V A L K

946 TCTTACGAGGAAGAGTTGGCGAAAGATCCACGTATTGCCGCCACT

S Y E E E L A K D P R I A A T

991 ATGGAAAACGCCCAGAAAGGTGAAATCATGCCGAACATCTCGCAG

M E N A Q K G E I M P N I S Q

1036 ATGTCCGCTTTCTGGTATGCCGTGCGTACTGCGGTGATCAACGCC

M S A F W Y A V R T A V I N A

1081 GCCAGCGGTCGTCAGACTGTCGATGCAGCCCTGAAAGACGCGCAG

A S G R Q T V D A A L K D A Q

1126 ACTAATTCGCCGTCCGGCGGCGAAAACCTGTATTTTCAGTCTGGA MBP end

T N S P S G G E N L Y F Q S G

1171 GGATCCCATATGATGGTGACCGGCTACCGGCTGTTCGAGGAGATT SmBiT

G S H M M V T G Y R L F E E I

1216 CTCGGGAGTTCCGGTGGTGGCGGGAGCGGAGGTGGAGGCTCGAGC GS-linker

L G S S G G G G S G G G G S S

1261 CGCATCCTAGGCGGAGTCATCAGCGCCATCAGCGAGGCGGCTGCG CAPNS1 start

R I L G G V I S A I S E A A A

1306 CAGTACAACCCGGAGCCCCCGCCCCCACGCACACATTACTCCAAC

Q Y N P E P P P P R T H Y S N

1351 ATTGAGGCCAACGAGAGTGAGGAGGTCCGGCAGTTCCGGAGACTC

I E A N E S E E V R Q F R R L

1396 TTTGCCCAGCTGGCTGGAGATGACATGGAGGTCAGCGCCACAGAA

F A Q L A G D D M E V S A T E

1441 CTCATGAACATTCTCAATAAGGTTGTGACACGACACCCTGATCTG

L M N I L N K V V T R H P D L

1486 AAGACTGATGGTTTTGGCATTGACACATGTCGCAGCATGGTGGCC

K T D G F G I D T C R S M V A

1531 GTGATGGATAGCGACACCACAGGCAAGCTGGGCTTTGAGGAATTC

V M D S D T T G K L G F E E F

1576 AAGTACTTGTGGAACAACATCAAAAGGTGGCAGGCCATATACAAA

K Y L W N N I K R W Q A I Y K

1621 CAGTTCGACACTGACCGATCAGGGACCATTTGCAGTAGTGAACTC

Q F D T D R S G T I C S S E L

1666 CCAGGTGCCTTTGAGGCAGCAGGGTTCCACCTGAATGAGCATCTC

P G A F E A A G F H L N E H L

1711 TATAACATGATCATCCGACGCTACTCAGATGAAAGTGGGAACATG

Y N M I I R R Y S D E S G N M

1756 GATTTTGACAACTTCATCAGCTGCTTGGTCAGGCTGGACGCCATG

D F D N F I S C L V R L D A M

1801 TTCCGTGCCTTCAAATCTCTTGACAAAGATGGCACTGGACAAATC

F R A F K S L D K D G T G Q I

1846 CAGGTGAACATCCAGGAGTGGCTGCAGCTGACTATGTATTCCTAA CAPNS1 end

Q V N I Q E W L Q L T M Y S *

**Table S7. Clustal multiple sequence alignment of CAPNS1, CAPN1, CAPN2 PEF domains.**

CAPNS1 RILGGVISAISEAAAQYNPEPPPPRTHYSNIEANESEEVRQFRRLFAQLAGDDMEVSATE 60

CAPN1 ---------------------------------SEEEIDENFKALFRQLAGEDMEISVKE 27

CAPN2 -------------------------------DISEDDIDDGFRRLFAQLAGEDAEISAFE 29

.*.: *: ** ****:* *:*. *

CAPNS1 LMNILNKVVTRHPDLKTDGFGIDTCRSMVAVMDSDTTGKLGFEEFKYLWNNIKRWQAIYK 120

CAPN1 LRTILNRIISKHKDLRTKGFSLESCRSMVNLMDRDGNGKLGLVEFNILWNRIRNYLSIFR 87

CAPN2 LQTILRRVLAKRQDIKSDGFSIETCKIMVDMLDSDGSGKLGLKEFYILWTKIQKYQKIYR 89

* .**.:::::: *:::.**.:::*: ** ::* * .****: ** **..*:.: *::

CAPNS1 QFDTDRSGTICSSELPGAFEAAGFHLNEHLYNMIIRRYSDESGNMDFDNFISCLVRLDAM 180

CAPN1 KFDLDKSGSMSAYEMRMAIESAGFKLNKKLYELIITRYSEPDLAVDFDNFVCCLVRLETM 147

CAPN2 EIDVDRSGTMNSYEMRKALEEAGFKMPCQLHQVIVARFADDQLIIDFDNFVRCLVRLETL 149

::* *:**:: : *: *:* ***:: :*:::*: *::: . :*****: *****:::

CAPNS1 FRAFKSLDKDGTGQIQVNIQEWLQLTMYS 209

CAPN1 FRFFKTLDTDLDGVVTFDLFKWLQLTMFA 176

CAPN2 FKIFKQLDPENTGTIELDLISWLCFSVL- 177

*: ** ** : * : .:: .** :::

* - exact identity

: - strongly conserved residues

. – weakly conserved residues

blank – no similarity
